# Supplementary material for: A voxel-based approach for simulating microbial decomposition in soil: Comparison with LBM and improvement of morphological models
Source: PLoS One. 2025 Mar 3;20(3):e0313853. doi: 10.1371/journal.pone.0313853 (PMC11875386; doi:10.1371/journal.pone.0313853)
Supplement: S1 File — (DOCX) [file pone.0313853.s001.docx]

S1 APPENDIX: Numerical schemes of the graph diffusion equation

Solving the graph diffusion equation (1) consists of solving the following set of equations (S1-1) for all nodes of the graph simultaneously,

$\forall i\in N,\frac{dm_{i}\left( t \right)}{dt}=\sum_{j:\left( i,j \right)\in E} -D_{DOM}\left( m_{i}\left( t \right)-m_{j}\left( t \right) \right)$ (S1-1)

Alternatively, we can solve the following equation

$\frac{dM\left( t \right)}{dt}=-D_{DOM}.\Delta M\left( t \right)$ (S1-2)

Where $M\left( t \right)=\left( m_{1}\left( t \right)...m_{n}\left( t \right) \right)$ and $\Delta=\left( \delta_{i,j} \right)_{1\leq i,j\leq n}$ is the Laplacian matrix of the graph $G\left( E,N \right)$ defined by:

$$\left\{ \begin{aligned} \begin{matrix} \delta_{i,j}=deg\left( i \right) & ifi=j \end{matrix} \\ \begin{matrix} \delta_{i,j}=-1 & ifi\neq j\wedge\left( i,j \right)\in E \end{matrix} \\ \begin{matrix} \delta_{i,j}=0 & otherwise \end{matrix} \end{aligned} \right.$$

where $deg\left( i \right)=\sum_{j:\left( i,j \right)\in E} 1$ is the number of adjacent nodes to the node i.

The equation S1-2 is an ordinary differential equation with analytical solution:

$$M\left( t \right)=e^{-D_{DOM}.t.\Delta}.M_{0}$$

To use this solution, we need to calculate the exponential of a large sparse matrix,

The matrix exponential $e^{-D_{DOM}.t.\Delta}$ of the matrix $-D_{DOM}.t.\Delta$ is computed as an infinite series:

$$I-D_{DOM}.t.\Delta+\frac{\left( D_{DOM}.t.\Delta\right)^{2}}{2!}-\frac{\left( D_{DOM}.t.\Delta\right)^{3}}{3!}+\ldots$$

Unfortunately, sparse matrices contain many zero elements, and efficient storage and computation leverage this sparsity. Our adjacency matrix $\Delta$ (of size tenth of millions, in the specific dataset of this study: 23 million) is very sparse as most nodes are only connected to a few other nodes (6 connected nodes as maximum, since we are using a 6-connectivity policy): the multiplication of sparse matrices often results in matrices with more non-zero elements, this is because new connections or interactions can emerge between previously disconnected nodes.

As higher powers of the matrix are computed (e.g., $\Delta^{2},\Delta^{3},\Delta^{4},\Delta^{5}$), the result becomes denser, leading to a rapid increase in memory and computational demands. Since the cost of calculating the analytical solution is high, we use Euler schemes to discretize the time derivative. However, there exist many approaches and tools capable of calculating the exponential of large sparse matrices like those found in MATLAB and SciPy. The existing methods can hardly be used, in practice, for matrices of size up to 20 million.

**Explicit scheme**

Let $i\in N$, The simplest way to discretize the first derivative with respect to time is by using the forward Euler time scheme, namely:

$$\frac{dm_{i}\left( t \right)}{dt}=\frac{m_{i}\left( t+\delta t \right)-m_{i}\left( t \right)}{\delta t}$$

Then we have:

$$\frac{{m_{i}}^{\left( k+1 \right)}-{m_{i}}^{\left( k \right)}}{\delta t}=\sum_{j:\left( i,j \right)\in E} D_{DOM}\left( {m_{i}}^{\left( k \right)}-{m_{j}}^{\left( k \right)} \right)$$

where $k$ is the iteration number (i.e., the discrete-time index), and $\delta t$ is the time step.

Equivalently, we have:

$${m_{i}}^{\left( k+1 \right)}={m_{i}}^{\left( k \right)}-\sum_{j:\left( i,j \right)\in E} D_{DOM}\delta t\left( {m_{i}}^{\left( k \right)}-{m_{j}}^{\left( k \right)} \right)$$

Rewritten compactly in matrix-vector form as follows,

$\begin{matrix} M^{\left( k+1 \right)}={\left( I-D_{DOM}\delta t\Delta\right)M}^{\left( k \right)} \\ QM^{\left( k \right)} \end{matrix}$ (Scheme 1)

where the matrix $Q=\left( q_{i,j} \right)_{1\leq i,j\leq n}$ is given by;

$$\left\{ \begin{aligned} \begin{matrix} q_{i,j}=1-D_{DOM}\delta tdeg\left( i \right) & ifi=j \end{matrix} \\ \begin{matrix} q_{i,j}=D_{DOM}\delta t & ifi\neq j\wedge\left( i,j \right)\in E \end{matrix} \\ \begin{matrix} q_{i,j}=0 & otherwise \end{matrix} \end{aligned} \right.$$

This approach is termed explicit because the update $M^{\left( k+1 \right)}$ is directly derived from $M^{\left( k \right)}$ through the application of the diffusion operator $Q$. The solution to the diffusion equation is obtained by iteratively applying the scheme 1 multiple times in succession, starting from an initial distribution $M^{\left( 0 \right)}$.

**Implicit scheme**

Let $i\in N$, another way to discretize the first derivative with respect to time is by using a backward Euler time scheme, namely:

$$\frac{{m_{i}}^{\left( k+1 \right)}-{m_{i}}^{\left( k \right)}}{\delta t}=\sum_{j:\left( i,j \right)\in E} -D_{DOM}\left( {m_{i}}^{\left( k+1 \right)}-{m_{j}}^{\left( k+1 \right)} \right)$$

Then we have:

$${m_{i}}^{\left( k \right)}={m_{i}}^{\left( k+1 \right)}+\sum_{j:\left( i,j \right)\in E} D_{DOM}\delta t\left( {m_{i}}^{\left( k+1 \right)}-{m_{j}}^{\left( k+1 \right)} \right)$$

Rewritten compactly in matrix-vector form,

$\begin{matrix} M^{\left( k \right)}={\left( I+D_{DOM}\delta t\Delta\right)M}^{\left( k+1 \right)} \\ BM^{\left( k+1 \right)} \end{matrix}$ (Scheme 2)

where the matrix $B=\left( b_{i,j} \right)_{1\leq i,j\leq n}$ is given by:

$$\left\{ \begin{aligned} \begin{matrix} b_{i,j}=1+D_{DOM}\delta tdeg\left( i \right) & ifi=j \end{matrix} \\ \begin{matrix} b_{i,j}={-D}_{DOM}\delta t & ifi\neq j\wedge\left( i,j \right)\in E \end{matrix} \\ \begin{matrix} b_{i,j}=0 & otherwise \end{matrix} \end{aligned} \right.$$

This scheme is referred to as implicit because it involves resolving a linear system to calculate the state $M^{\left( k+1 \right)}$ based on $M^{\left( k \right)}$, which necessitates the inversion of the matrix $B$.

In practice, rather than exact inversion, a few iterations of a linear solver are commonly employed in place of inverting the matrix.

Given that $B$ is a large, sparse, and symmetric positive definite matrix, the conjugate gradient method is the optimal iterative solver for addressing scheme 2. The conjugate gradient method is particularly effective for symmetric positive definite matrices, as it leverages conjugate directions to minimize the error between the candidate solution and the target iteratively. In [20], to solve an implicit scheme for simulating diffusion using Fick's first law of diffusion in a graph of connected spheres approximating the pore space, the conjugate gradient method was employed. This method has shown advantages in scenarios involving large-scale, sparse linear systems, with its efficiency further enhanced when a suitable preconditioner is employed.

**S2 APPENDIX: Microbial decomposition simulations : tests on time steps**

**Table S2T1: Tests of VGA for simulation of microbial decomposition of organic matter**

| **Test** | | **1** | **2** | **3** | **4** |
| --- | --- | --- | --- | --- | --- |
| **Figure** | | **S2A** | **S2B** | **9** | **S2C** |
| **Time step diffusion** | **Explicit** | **-** | **0.1 (s)** | **0.1 (s)** | **-** |
|  | **Implicit** | **1 (s)** | **-** | **-** | **5 (s)** |
| **Time step transformation** | | **1 (s)** | **0.1 (s)** | **0.43 (s)** | **5 (s)** |
| **Computing time** | | **5.3 (day)** | **7.9 (day)** | **6.4 (day)** | **3.2 (day)** |

Figure S2A: LBM-based approach using synchronous transformation with a time step of 0.43s, PNGM-based method using asynchronous transformation with 5s time step, VGA using implicit scheme and asynchronous transformation using same time step 1s.


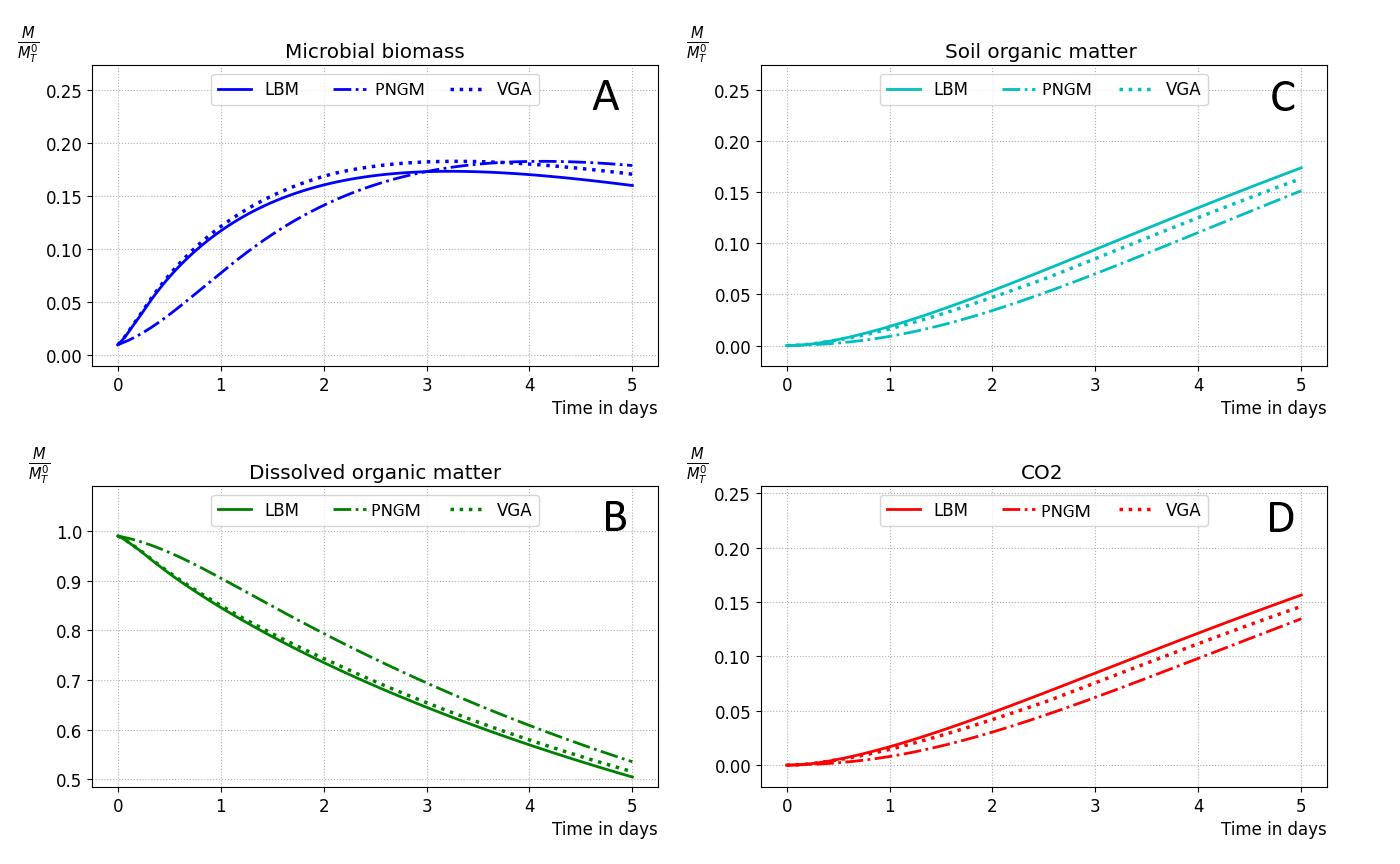


Figure S2B: LBM-based approach using synchronous transformation with a time step of 0.43s, PNGM-based method using asynchronous transformation with 5s time step, VGA using explicit scheme and asynchronous transformation using same time step 0.1s.


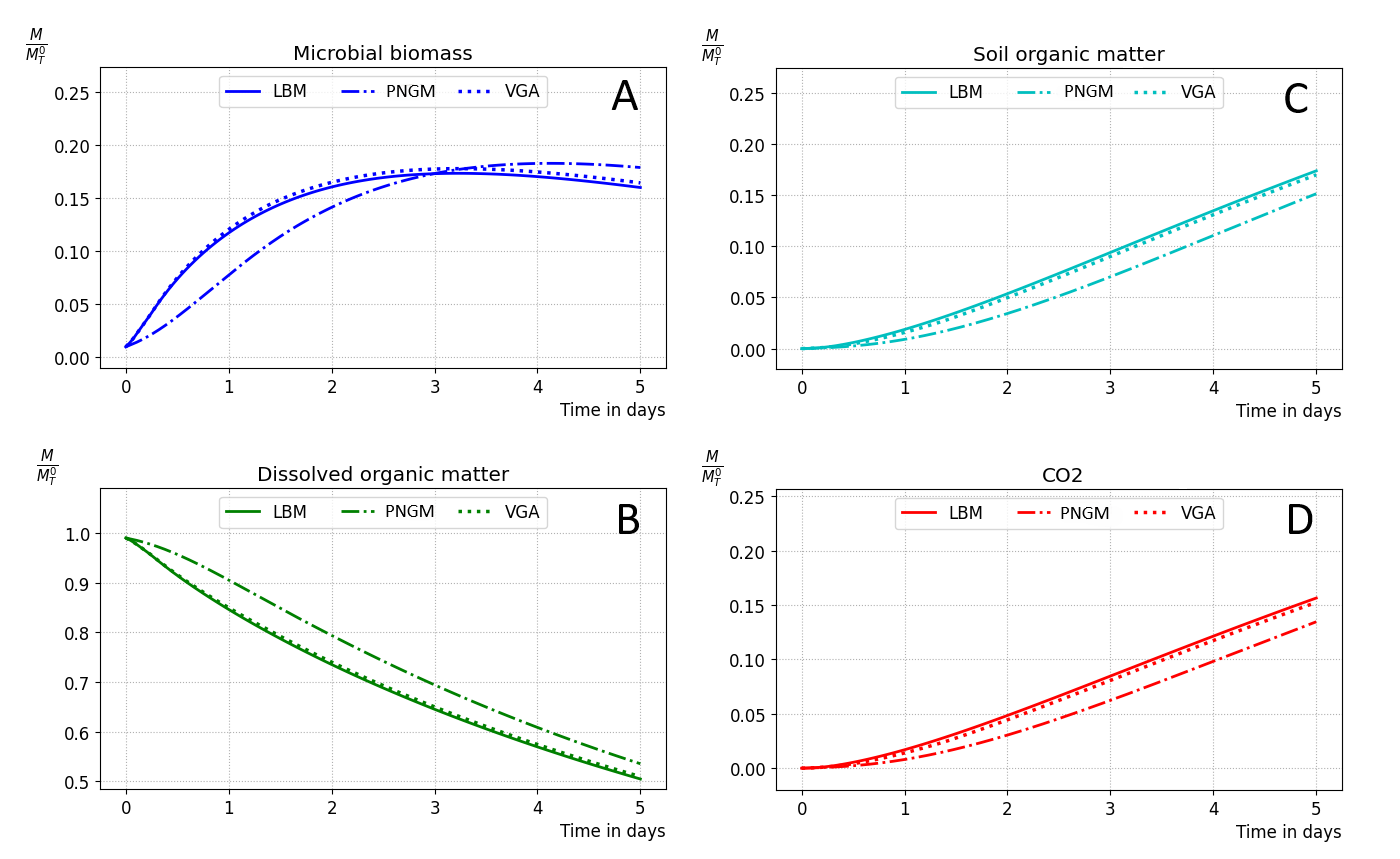


Figure S2C: Microbial decomposition simulation: LBM-based approach using synchronous transformation with a time step of 0.43s, PNGM-based method using asynchronous transformation with a 5s time step, VGA using implicit scheme and asynchronous transformation using same time step of 5s.


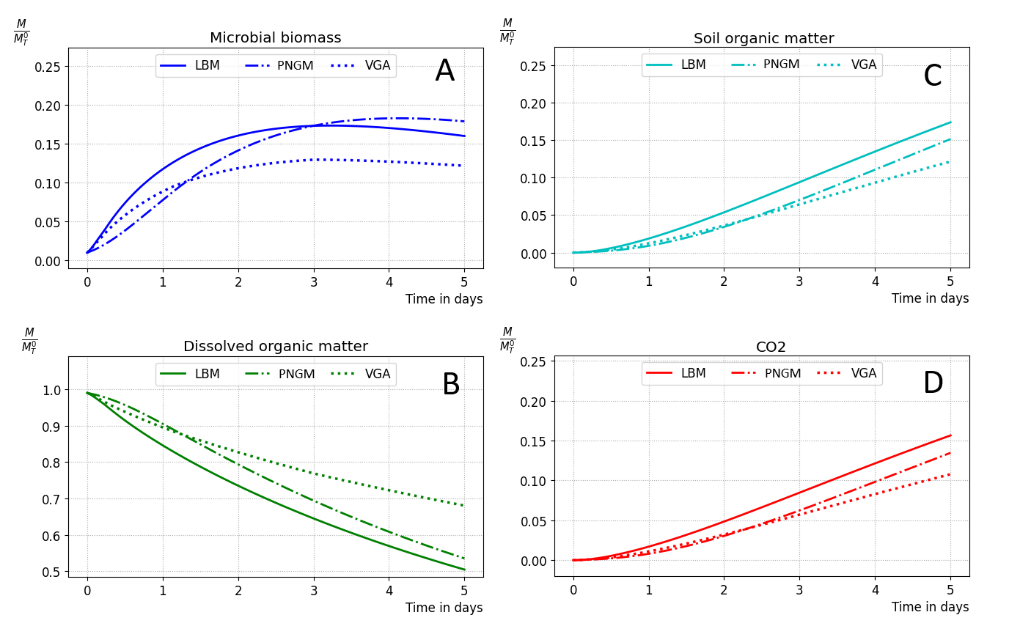


S3 APPENDIX: Synchronous and Asynchronous schemes

S3A - Flowchart 1: Synchronous algorithm of transformation processes

*
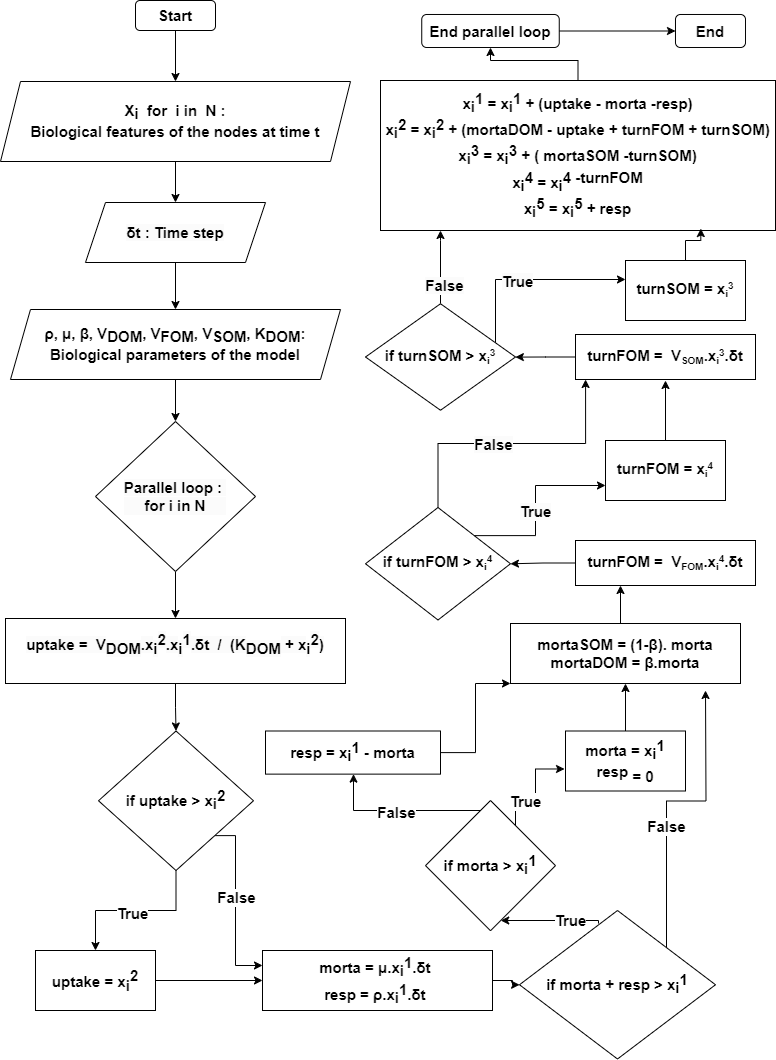
*

S3B - Flowchart 2: Asynchronous algorithm of transformation processes


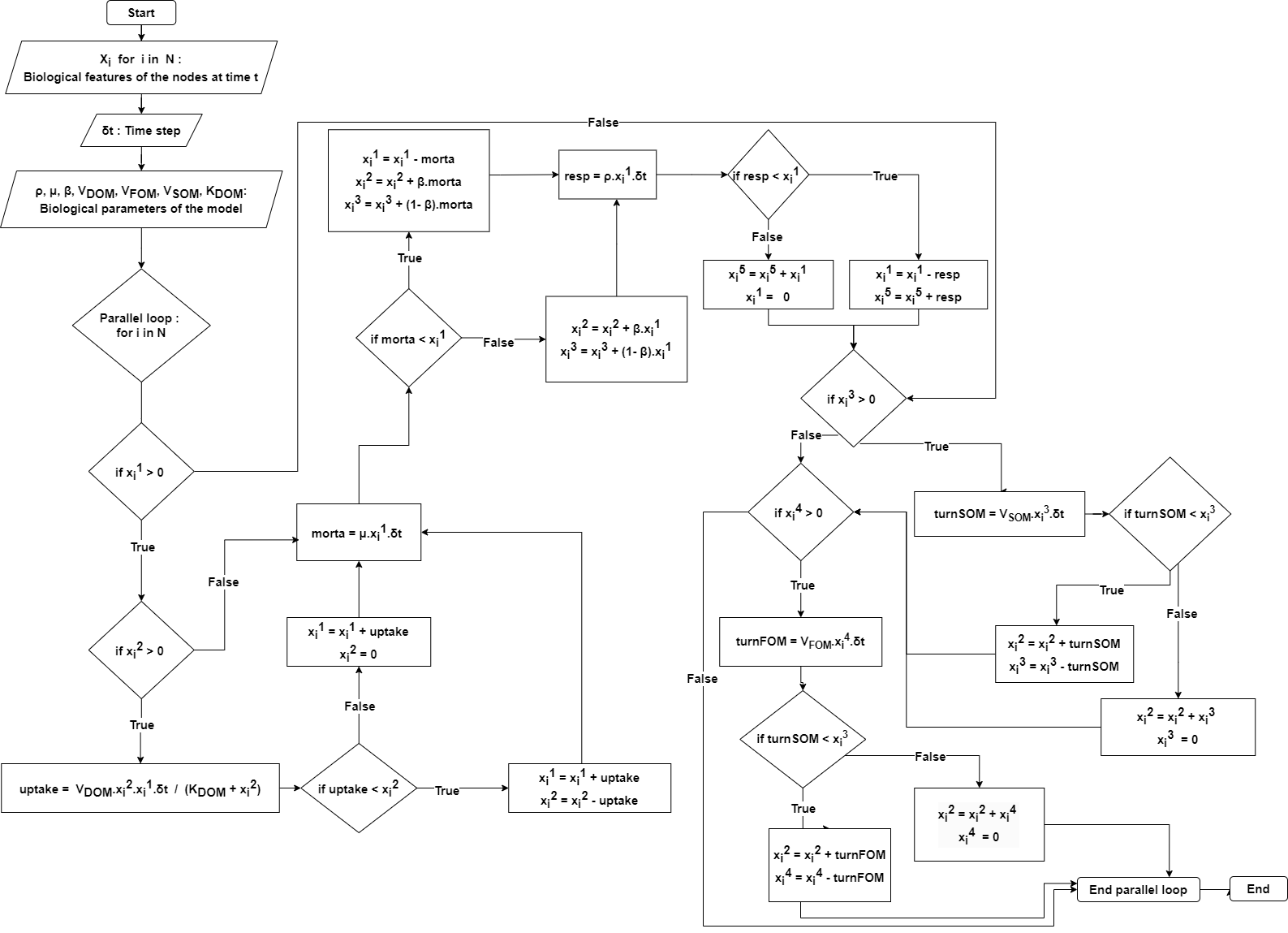


Flowcharts S3A and S3B provide a stable algorithm to approximate dynamics of equation (12), by tolerating the use of reasonable time steps. In contrast, applying drastically the explicit scheme (in a synchronous way) of transformation (equation 13) would require using very small-time steps, that would increase computing time
