# Supplementary material for: A voxel-based approach for simulating microbial decomposition in soil: Comparison with LBM and improvement of morphological models
Source: PLoS One. 2025 Mar 3;20(3):e0313853. doi: 10.1371/journal.pone.0313853 (PMC11875386; doi:10.1371/journal.pone.0313853)
Supplement: Attachment — Submitted filename: Review_Report_PONE-D-24-24748.pdf [file pone.0313853.s002.pdf]

A Voxel-based Approach for Simulating Microbial Decomposition in Soil: Comparison with LBM and Improvement of Morphological Models

The authors investigated a voxel-based approach for simulating microbial decomposition in soil: comparison with LBM and improvement of morphological models. **Before the publication of this paper, I have raised some issues as follows:**

Major issues:

1. The authors should improve the **Abstract** of the study.
2. The authors should revise the **Introduction** of the study by including the novelty.
3. What was the basis for choosing the mentioned ranges for the investigated parameters?
4. The authors should thoroughly clarify each concept with physical significance.
5. A comparison is expected with the experimental results.
6. When utilizing articles in sentences, authors should be more careful.
7. A reasonable physical explanation should be provided for the observed trends, not only to report what is graphically seen in the figures. More physical insight into the Discussion section is needed.
8. Too many passive sentences are in this article.
9. The conclusions must be More Specific to the obtained results. The present conclusions are more general.
10. The authors should write the practical applications and advantages of this study.

Minor issues:

Abstract:

Page 1

Line 19: "matter," It seems that an unnecessary comma. Consider removing the comma.

Line 28: "simulation," It appears that you have an unnecessary comma in a compound predicate. Consider removing it.

Line 29: "Mosaic" There seems to be a problem with article usage here.

Introduction :

Page 2

Line 43: "to fully capture its complexity" The to-infinitive **to capture** has been split by the modifier **fully**. Avoiding split infinitives can help your writing sound more formal.

Line 45: "microscopy" It seems that you are missing a comma. Consider adding a comma.

Line 49: It appears that **starting** may be unnecessary in this sentence. Consider removing it

Line 52: It appears that the preposition **in** is redundant. Consider removing it.

Page 3

Line 58: It seems that **LBM based** is missing a hyphen. Consider adding the hyphen(s).

Line 60: It seems that the verb **require** does not agree with the subject. Consider changing the verb form.

Line 61: It seems that **real time** is missing a hyphen. Consider adding the hyphen(s).

Line 67: The word **especially** is often overused. Consider using a more specific synonym to improve the sharpness of your writing.

Line 72: "voxels," It seems that you have an unnecessary comma. Consider removing the comma.

Page 3

Line 91: "(100% saturation),**"** It appears that you have an unnecessary comma in a compound object. Consider removing it.

Page 6

Line 138: The word **apply** appears repeatedly in this text. Consider using a synonym in its place.

Line 160: "diffusion" It seems that there is an article usage problem here.

Page 7

Line 162: The phrase **is equal to** maybe wordy. Consider changing the wording.

Page 11

Line 242: The phrase **stands out as** may be wordy. Consider changing the wording.

Page 12

Line 256: The word **respire** doesn't seem to fit this context. Consider replacing it with a different one.

Page 15

Line 305: This sentence **"as well as"** may contain incorrect or awkward phrasing. Consider changing it.

Page 16

Line 331: "then" It seems that you are missing a comma. Consider adding a comma.

Line 332: "S1" It seems that there is an article usage problem here.

Page 17

Line 339: The word **involves** appears repeatedly in this text. Consider using a synonym in its place.

Line 349: "and" It seems that you are missing a comma. Consider adding a comma.

Page 18

Line 365: It appears that your sentence or clause uses an incorrect form of the verb **resulted**. Consider changing it.

Line 368: "MATLAB" It seems that there is an article usage problem here.

Page 19

Line 382: "Voxel" It seems that there is an article usage problem here.

Line 385: It appears that you are missing a comma before the coordinating conjunction **and** in a compound sentence. Consider adding a comma.

Line 386-387: A knowledgeable audience might find this sentence hard to read. Consider breaking it into two.

Line 400: It appears that your sentence or clause uses an incorrect form of the verb **distribute**. Consider changing it**.**

Page 20

Line 406: The phrase **in accordance with** may be wordy. Consider changing the wording.

Line 407: "that we" It seems that there is a pronoun problem here.

Line 417: It appears that **results** may be unnecessary in this sentence. Consider removing it.

Page 21

Line 427: The word **larger** is often overused. Consider using a more specific synonym to improve the sharpness of your writing.

Line 427: The phrase **illustrates a comparison of** may be wordy. Consider changing the wording.

Line 432: "method," It seems that you have an unnecessary comma. Consider removing the comma.

Line 433: "LBM-based" It seems that there is an article usage problem here.

Line 434: "PNGM-based" It seems that there is an article usage problem here.

Page 22

Line 460-461: It seems that **LBM based** is missing a hyphen. Consider adding the hyphen(s).

Line 461: "diffusion,**"** It appears that you have an unnecessary comma in a compound object. Consider removing it.

Line 462: "same" It seems that there is an article usage problem here.

Page 34

Line 679: "stochastic" It seems that preposition use may be incorrect here.

Line 680: "normalized" It seems that there is an article usage problem here.

Line 681: "update" It appears that your sentence or clause uses an incorrect form of the verb **update**. Consider changing it.

Page 35

Line 697: The phrase **compared to** may be wordy. Consider changing the wording.

Line 698: The phrase **Accurate determination of** may be wordy. Consider changing the wording.

Page 36

Line 720: The noun phrase **explicit scheme** seems to be missing a determiner before it. Consider adding an article.

Line 720: "step," It appears that you have an unnecessary comma in a compound object. Consider removing it.

Line 726: It appears that **from** may be unnecessary in this sentence. Consider removing it.

Conclusion:

Page 38

Line 759: The word **huge** is often overused. Consider using a more specific synonym to improve the sharpness of your writing.

Line 761: "an enhanced" The indefinite article, **an**, may be redundant when used with the uncountable noun **accuracy** in your sentence. Consider removing it.

Line 765: It seems that the verb **outperform** does not agree with the subject. Consider changing the verb form.

Line 769: It appears that **hence** may be unnecessary in this sentence. Consider removing it.
