# Supplementary material for: A voxel-based approach for simulating microbial decomposition in soil: Comparison with LBM and improvement of morphological models
Source: PLoS One. 2025 Mar 3;20(3):e0313853. doi: 10.1371/journal.pone.0313853 (PMC11875386; doi:10.1371/journal.pone.0313853)
Supplement: Attachment — Submitted filename: Response_to_Reviewers_auresp_2.docx [file pone.0313853.s005.docx]

Reviewers' comments:

Reviewer's Responses to Questions

**Comments to the Author**

1. If the authors have adequately addressed your comments raised in a previous round of review and you feel that this manuscript is now acceptable for publication, you may indicate that here to bypass the “Comments to the Author” section, enter your conflict of interest statement in the “Confidential to Editor” section, and submit your "Accept" recommendation.

Reviewer #1: All comments have been addressed

Reviewer #3: (No Response)

2. Is the manuscript technically sound, and do the data support the conclusions?

The manuscript must describe a technically sound piece of scientific research with data that supports the conclusions. Experiments must have been conducted rigorously, with appropriate controls, replication, and sample sizes. The conclusions must be drawn appropriately based on the data presented.

Reviewer #1: Yes

Reviewer #3: Yes

3. Has the statistical analysis been performed appropriately and rigorously?

Reviewer #1: YesReviewer #3: N/A

4. Have the authors made all data underlying the findings in their manuscript fully available?

The [PLOS Data policy](http://c05y1x9s.r.us-east-2.awstrack.me/L0/http:%2F%2Fwww.plosone.org%2Fstatic%2Fpolicies.action%23sharing/1/010f0191fce06a9f-5f19d548-1c5a-456b-b624-68df4404bf3d-000000/cUuCw4Fe-T9Iy0qtMqPyiycblG8=176) requires authors to make all data underlying the findings described in their manuscript fully available without restriction, with rare exception (please refer to the Data Availability Statement in the manuscript PDF file). The data should be provided as part of the manuscript or its supporting information, or deposited to a public repository. For example, in addition to summary statistics, the data points behind means, medians and variance measures should be available. If there are restrictions on publicly sharing data—e.g. participant privacy or use of data from a third party—those must be specified.

Reviewer #1: Yes

Reviewer #3: No

In line 313, we included a link to the C implementation for VGA and for approximating diffusional conductance coefficients.

We also included a link to the 3D image used in the study in line 314.

5. Is the manuscript presented in an intelligible fashion and written in standard English?

PLOS ONE does not copyedit accepted manuscripts, so the language in submitted articles must be clear, correct, and unambiguous. Any typographical or grammatical errors should be corrected at revision, so please note any specific errors here.

Reviewer #1: Yes

Reviewer #3: Yes

6. Review Comments to the Author

Please use the space provided to explain your answers to the questions above. You may also include additional comments for the author, including concerns about dual publication, research ethics, or publication ethics. (Please upload your review as an attachment if it exceeds 20,000 characters)

Reviewer #1: The authors have adequately addressed your comments raised in a previous round of review and you feel that this manuscript is now acceptable for publication.

Reviewer #3: I appreciate that author's efforts and think that most of the comments have been addressed adequately. However, changes in the manuscript are unfortunately not indicated everywhere in a way that allows to properly check if the claimed response is taken up in the manuscript (e.g., by providing line numbers of the new manuscript in the response).

We apologize for the oversight. In appendix to this itemized response to the Reviewer’s comments, we have included our former itemized response now completed with the line numbers of the revised manuscript.

I found some critical points regarding the abstract where I see a strong discrepancy between the claimed improvement and the actual revision of the manuscript. Since the author's responses are not fully trackable in the adapted manuscript for me, I have to consider the provided response to reviewers and the manuscript revision as insufficient and cannot recommend to publish the manuscript in the current version without clarifying the remaining issues.

\# Abstract

In their response, the authors claim that aims and objectives are now clearly stated. However, I cannot find such a statement in the abstract and the authors also missed to indicate with specific line numbers which part of the revised text exactly provides aims and objectives of the study. The added sentence "The research gap is to propose forward and backward Euler discretization scheme to simulate joined diffusion and transformation processes within the context of microbial decomposition" needs to be clarified. Why is it a research gap to propose a discretization scheme? In my understanding that's a technical detail of the proposed approach but not a research gap.

We have added the following in the beginning of the abstract in lines 16-19:

which now states clearly the aim and objective of the paper

“This paper deals with the computational modeling of biological dynamics in soil using an exact micro-scale pore space description from 3D Computed Tomography (CT) images. Within this context, computational costs and storage requirements constitute critical factors for running simulations on large datasets over extended periods.”

.

I could further not find the claimed clarification on the two-phase design of the model.

Although the model is suited for three-phase system (see in Monga et al., 2008), we decided to remove this part as it is not central to the VGA method and simply acknowledge fully saturated conditions (lines 19-22) :

“In this research, we represent the pore space by a graph of voxels (Voxel Graph-Based Approach, VGA) and model transport in fully saturated conditions (two-phase system) using Fick’s law and coupled diffusion with biodegradation processes to simulate microbial decomposition in soil.”

Also in material and methods section we mention how our VGA can be used to simulate diffusion and biodegradation in partially saturated medium after a drainage method (lines 108-115)

Additionally, the revised abstract has been further extended and should be significantly shortened by removing details.

We have shortened the abstract.

\# Introduction

At the end of the revised introduction, the authors give a kind second abstract describing the approach and study results in quite some detail (l 99ff in the revised manuscript with track changes). This part should be strongly condensed as it is still anticipating results.

We have removed all anticipating results. The changes can be found in lines 77-90.

\# Material and Methods

The authors state "In the revised manuscript, we have mentioned this point more clearly." Where exactly? The authors respond the following: "Moreover, directly using the analytical solution for simulations is complex because it would need to be coupled with the transformation processes at each simulation step which is not straightforward. While using the analytical solution would be preferable for diffusion alone, its application becomes challenging in our context, as it requires calculating the exponential of a large matrix, introducing additional computational difficulties."

In my understanding this response does not provide a convincing argument. For me and also for readers it remains unclear why calculating the exponential of a matrix is more computational demanding compared to numerically solving a differential equation.

As mentioned in the revised answers of the first review (in annex to this review), and now included in the text (supplementary material S1 lines 886-908):

“Directly using the analytical solution for simulations is complex because it requires calculating the exponential of a large matrix, introducing additional computational difficulties.

Indeed, the equation of diffusion is the following

$\frac{dM\left( t \right)}{dt}=-D_{DOM}.\Delta M\left( t \right)$	(S1-2)

Where $M\left( t \right)=\left( m_{1}\left( t \right)...m_{n}\left( t \right) \right)$ and $\Delta=\left( \delta_{i,j} \right)_{1\leq i,j\leq n}$ is the Laplacian matrix of the graph $G\left( E,N \right)$ defined by:

$$\left\{ \begin{aligned} \begin{matrix} \delta_{i,j}=deg\left( i \right) & ifi=j \end{matrix} \\ \begin{matrix} \delta_{i,j}=-1 & ifi\neq j\wedge\left( i,j \right)\in E \end{matrix} \\ \begin{matrix} \delta_{i,j}=0 & otherwise \end{matrix} \end{aligned} \right.$$

where $deg\left( i \right)=\sum_{j:\left( i,j \right)\in E} 1$ is the number of adjacent nodes to the node i.

the equation S1-2 is an ordinary differential equation with analytical solution:

$$M\left( t \right)=e^{-D_{DOM}.t.\Delta}.M_{0}$$

To use this solution, we need to calculate the exponential of a large sparse matrix,

The matrix exponential $e^{-D_{DOM}.t.\Delta}$ of the matrix $-D_{DOM}.t.\Delta$ is computed as an infinite series:

$$I-D_{DOM}.t.\Delta+\frac{\left( D_{DOM}.t.\Delta\right)^{2}}{2!}-\frac{\left( D_{DOM}.t.\Delta\right)^{3}}{3!}+\ldots$$

Unfortunately, sparse matrices contain many zero elements, and efficient storage and computation leverage this sparsity. Our adjacency matrix $\Delta$ (of size tenth of millions, in the specific dataset of this study: 23 million) is very sparse as most nodes are only connected to a few other nodes (6 connected nodes as maximum, since we are using a 6-connectivity policy): the multiplication of sparse matrices often results in matrices with more non-zero elements, this is because new connections or interactions can emerge between previously disconnected nodes.

As higher powers of the matrix are computed (e.g., $\Delta^{2},\Delta^{3},\Delta^{4},\Delta^{5}$), the result becomes denser, leading to a rapid increase in memory and computational demands.

Since the cost of calculating the analytical solution is high, we use Euler schemes to discretize the time derivative.

However, there exist many approaches and tools capable of calculating the exponential of large sparse matrices like those found in MATLAB and SciPy. The existing methods can hardly be used, in practice, for matrices of size up to 20 million”

The rationale for decoupling growth from respiration is unfortunately not convincing to me and should be clearly outlined for readers in the manuscript. I checked the Vogel et al. (2015) publication, but could also not find a convincing rationale to decouple growth and growth respiration in that publication.

Thank you for this in-depth comment. As stated in Vogel et al., 2015, we followed the approach of the SOMKO model (Gignoux et al., 2001) and MIOR model (Masse et al., 2007) where microbes are subjected to 3 physiological processess: survival, maintenance and growth. In this approach, CO2 flux is computed as a linear function of biomass. This approach considers offer-demand issue: indeed, in case of an offer of C too low to sustain maintenance, growth (demand) is not fulfilled. As reported in the review of Manzoni & Porporato (2009) and raised by the reviewer, most of the C models assume growth respiration proportional to uptake (yield or carbon use efficiency CUE approach). In these models, growth respiration is thus not included in the carbon mass balance to consider offer-demand issue. In more complex approaches, biosynthesis and CO2 flux are calculated from the stoechiometry of chemical reactions (e.g. Ginovart et al., 2005).

References:

Bölscher, T., Vogel, C., Olagoke, F.K., Meurer, K.H., Herrmann, A.M., Colombi, T., et al., 2024. Beyond growth: the significance of non-growth anabolism for microbial carbon-use efficiency in the light of soil carbon stabilisation. Soil Biol. Biochem. 109400.
Gignoux, J., House, J., Hall, D., Masse, D., Nacro, H.B., Abbadie, L., 2001. Design and test of a generic cohort model of soil organic matter decomposition: the SOMKO model. Global Ecology and Biogeography 10, 639–660.
Ginovart, M., Lopez, D., Gras, A., 2005. Individual-based modelling of microbial activity to study mineralization of C and N and nitrification process in soil. Nonlinear Analysis – Real World Applications 6, 773–795.
Manzoni, S., Capek, P., Porada, P., Thurner, M., Winterdahl, M., Beer, C., Brüchert, V., Frouz, J., Herrmann, A.M., Lindahl, B.D., Lyon, S.W., Šantrucková, H., Vico, G., Way, D., 2018. Reviews and syntheses : carbon use efficiency from organisms to ecosystems – definitions, theories, and empirical evidence. Biogeosciences 15, 5929–5949. <https://doi.org/10.5194/bg-15-5929-2018>.
Manzoni, S., Porporato, A., 2009. Soil carbon and nitrogen mineralization: Theory and models across scales. Soil Biology & Biochemistry 41, 1355-1379.
Masse, D., Cambier, C., Brauman, A., Sall, S., Assigbetse, K., Chotte, J.L., 2007. MIOR: an individual-based model for simulating the spatial patterns of soil organic matter microbial decomposition. European Journal of Soil Science 58, 1127–1135.

These clarifications have been added to the text in lines 248-251.

**Appendix presenting the revised itemized response of the former Reviewers comment scompleted with line numbers in the revised manuscript:**

**Reviewer #1:**

This study presents a novel computational method for simulating microbial decomposition in soil using 3D micro-CT images. It uses a valuated graph of connected voxels to model complex diffusion and transformation processes. The method includes parallelization strategies and evaluates various numerical techniques. Validation against LBioS (lattice-Boltzmann method) and Mosaic (Pore Network Geometrical Modelling) shows it matches LBM-based simulation accuracy with a quarter of the computing time and exceeds Mosaic in accuracy without calibration. Additionally, it enhances PNGM-based simulations by approximating diffusional conductance coefficients using stochastic gradient descent.

Based on the initial assessment of this manuscript, the current decision is a major revision to reconsider the publication with Plos One.

Abstract1. Line 18-19: It would be good to point out the research gap before running into aims at the beginning of your abstract (be concise, please; after all, it is just an abstract)

We added a few sentences describing the research gap and the aims of the paper in lines 16 to 19.

.2. Line 25: Could you please define LbioS in your abstract as it first appears in your content?

We defined "LBioS" in the abstract in lines 26–27.

3. Line 28: one-fourth -> a quarter

We replaced "one-fourth" with "a quarter" in line 29.

4. Line 33: It would be better if you could restate/highlight your engineering contribution from the novel method you developed in this study (please be concise; after all, it is just an abstract)

We described the engineering contribution of our method in lines 19-25.

#Introduction

5. Line 51: These efforts aim to … ? or These studies aim to … ? Can efforts aim to do something?

We changed the sentence "These efforts aim to..." to "These works aimed to..." in line 52.

6. Line 68: You have not accomplished your literature review by identifying a research gap in high clarity. You have not compared and contrasted the pros and cons from the historical track of previous studies (the initial, later progress, and state of the art by now; what has been done already and what else has not been achieved yet?) in order to identify those research gaps before you propose your research objectives, followed by methods from Line 69. Your review of published works should solidly support your research purposes before heading into it. There is a significant gap or logical disconnection between Lines 68 and 69.

We have restructured the literature review to more clearly contrast previous studies, identify gaps, and establish the rationale for our proposed research objectives and methods. These changes are reflected in lines 50–79.

7. Lines 82-87: You have structurally illustrated your paper in the second last paragraph without mentioning the specific sections/chapters. You don’t need to redo it with an independent paragraph that looks slightly redundant.

We have revised the second-to-last paragraph (lines 84-90) to include a brief mention of the key sections without adding a separate paragraph.

8. At the end of your Introduction and right after your research proposal, you should re-highlight your research significance and engineering contributions to dazzle the reviewers and readers, who have short memories after reading a series of lengthy content, because not all of them can easily recall all the content you provided in the background and literature review in the Introduction. Note that it must be concise and informative.

We have revised the conclusion of the introduction (lines 84-90)

Material and methods

9. Lines 91-92: You can just write, “In this study, we only considered the condition that soil is fully saturated, i.e., soil pore space is filled with water, in exception that soil under the unsaturated condition, i.e., soil void space is partially filled with soil moisture.” An aquatic environment could represent many other meanings, not only in topsoil, vadose zone, and groundwater under the phreatic line (i.e., groundwater table) but also in other water bodies, e.g., nearshore seawater, surface flow, etc.

Thank you for the clarification. We have revised the sentence as suggested to more accurately describe the fully saturated soil condition while avoiding ambiguity regarding the term "aquatic environment." The changes have been made in lines 94 to 95.

10. For a numerical contribution like this, I recommend you relocate your pseudo code (algorithm) into an appendix and leave a Matlab or Python code in your supplementary file so many researchers interested in this code will further work on it. It would be much better if you could draw a flow chart for your pseudo-code as a figure in the main text rather than in the appendix and supplementary material.

We appreciate your suggestion. We have replaced the pseudocodes with flowcharts in lines 988-992, which we believe makes the process more readable and visually intuitive.

Additionally, in section 2.4 (Implementation Details), we have included, in line 313, a link to the C implementation for VGA and for approximating diffusional conductance coefficients.

We include also a link to the 3D image used in the study in line 314.

11. Since it was not observable in your figures in terms of a few very important key points for a numerical simulation, e.g., boundary conditions, initial conditions, etc., you should better visualize those in your simulation demo figures and also provide other information regarding convergence and numerical stability issues (e.g., size of discretization, temporal step in your implicit and explicit schemes, simulating scale compared to single particles or the representativeness of REV, etc.) in the numerical method you just developed. Those will help you defend your work from other peer reviewers who are experts in developing numerical schemes.

Thank you for highlighting the importance of including more details regarding boundary conditions, initial conditions, and key numerical aspects such as discretization size, time-stepping, and representativeness.

Boundary Conditions: These are now detailed in subsection 2.6.3 of the paper, in lines 450 to 453.

Initial Conditions: These are explained in the Results and Discussion section. Lines 492 to 518 cover the initial conditions related to diffusion, and Lines 550 to 559 describe the initial conditions related to microbial decomposition.

Stability and Convergence: Stability results are discussed in the Results and Discussion section. Additionally, we have included explicit details about numerical stability and convergence in subsection 2.2.2, found in lines 202 to 206. Briefly:

- Explicit Scheme: Easier to implement and computationally cheaper but requires smaller time steps to maintain stability.
- Implicit Scheme: More stable with respect to time step size but involves solving linear systems at each time step.

Results and discussion

12. As you used image analysis on CT scans, have you provided adequate technical details on CT scanning and image processing, e.g., voxel-length calibration, ratio, measuring precision, etc.?

We have provided additional context and details about the 3D microcomputed tomography (µCT) image data, soil characteristics, and the segmentation techniques in lines 442 to 448.

13. Line 516: You claimed you adopted the D3Q9 scheme for LBM simulation. So far, to the best of my knowledge, it should have D2Q9, D3Q15, and D3Q27. Could you please visualize your D3Q9 cell in 3D space, as you demonstrated in Figure 5? Please don’t forget to update all figure numbers in both their captions and content.

Thank you for the feedback. It is true that, by mistake, we referenced the D3Q9 scheme instead of D3Q19. We have updated the manuscript accordingly in line 612.

Below is a figure illustrating the D3Q19 scheme used in this study, sourced from: https://link.springer.com/article/10.1007/s11242-011-9914-7.

14. As for many diffusion coefficients or diffusivities you adopted in your numerical simulations, I recommend you defend your selection of those by comparing them against published works, i.e., necessary citations of new references should be added in. This question could also be raised to select other parameters of the properties of fluid and porous media in your work. It will convince experts who have more experience in this area.

Thank you for your feedback on the selection of diffusion coefficients and parameters. We adopted these values from previous studies to ensure they remained within experimentally relevant ranges (lines 572 to 575). While diffusion coefficients can vary, our simulations show consistent trends, indicating that the results are robust and not dependent on exact values. We have clarified this in the revised manuscript and discussed in Results and Discussions section how our findings reflect general behavior rather than sensitivity to specific parameters.

15. Lines 734-754: The pros and cons could be better summarized in a table for comprehensive comparison and contrast. You can also do so to compare different numerical methods. It will shorten the content you must deliver to readers and make the paper more concise and informative, i.e., straightforward to read and check all essentials in just a few tables.

Thank you for your insightful suggestion regarding the organization of the content. In response, we have added a table (Table 1, lines 711 to 713) that outlines the pros and cons of the different approaches, offering a clear and comprehensive comparison.

Conclusions

16. Please reconstruct your concluding section as follows: Paragraph 1: Concise summary of your research gaps identified through literature review and research objectives; Briefing your methodology with some very critical settings; Paragraph 2: Listing your conclusions or research findings point-by-point; Paragraph 3: Mention the limitations and reflection of your work; Highlight the significance of your research and engineering applications (this should be done at the end of your Introduction as well because it will substantially enhance your academic significance, i.e., self-advertising).

Thank you for your valuable feedback.

We have revised the conclusion to include the research gaps, objectives, and a summary of the methodology (lines 717 to 727).

The findings and contributions are listed in the following lines 728-735.

We concluded with a discussion of the advantages, limitations, and significance of the research, including its practical applications (lines 736 to 765).

References

17. The reference list you provided is inconsistent; e.g., some have the year in brackets ahead while others are at the end with even the month. Please sort those out and get them fixed according to the PloS One standard.

Thank you for pointing out the inconsistencies in the reference list.

We applied Vancouver formatting to all the references and added some necessary ones in the revised manuscript (lines 773 to 878).

Figures and Quality

18. All figures are provided in very blurry forms, resulting in difficulty in reading and checking all essential details. It could be an issue caused by Word-to-PDF transformation. Moreover, despite Figures 1-5, the font sizes of values in axes, labels, and legends in all other figures are too small to be read; e.g., the axis label “z” is missed in Figure 6 without any unit and interpretation.19. Last but not least, please scrutinize your manuscript using grammar software (e.g., Grammarly, Writefull, Quiltbot, etc.) to screen out all grammatical mistakes and typos before the subsequent submission of your revised draft.

Thank you for your feedback regarding the figures. We have updated the figures to ensure they are of the highest quality. Additionally, we have corrected Figure 6 (lines 469-473) to include the missing axis label "z."

**Reviewer #2:**

The authors investigated a voxel-based approach for simulating microbial decomposition in soil: comparison with LBM and improvement of morphological models. Before the publication of this paper, I have raised some issues as follows:

Major issues:

1. The authors should improve the Abstract of the study.

We have updated the abstract to make it clearer and more concise, explicitly stating the aim and objectives of the paper from lines 16 to 19, as well as the contributions of our study from lines 19 to 25.

2. The authors should revise the Introduction of the study by including the novelty.

We clearly highlight the novelty of our approach in the introduction in lines 77-83.

3. What was the basis for choosing the mentioned ranges for the investigated parameters?

Parameterization of our model has been performed on experimental measurements of growth and respiration curves (Monga et al., 2014) of Arthrobacter i.e. cultivated in optimized substrate conditions. In this respect, we followed the classical calibration approach done for years to parameterize the CUE value. Doing so means also that our respiration rate value lumps together maintenance (renewal of biomass synthesis) and growth (new biomass synthesis) respiration. This information can be found in lines 572-575.

4. The authors should thoroughly clarify each concept with physical significance.

We have included detailed explanations of the underlying physical principles.

5. A comparison is expected with the experimental results.

We agree that comparing our results with experimental data is important.

This paper primarily aims to evaluate our simulation method against existing approaches. However, the model used in this study was validated by experimental results in Monga et al. (2014), as cited in line 572.

6. When utilizing articles in sentences, authors should be more careful.

Thank you for pointing this out. We have carefully reviewed and revised the use of articles in sentences usage throughout the manuscript.

7. A reasonable physical explanation should be provided for the observed trends, not only to report what is graphically seen in the figures. More physical insight into the Discussion section is needed.

We revised the results to include more physical explanations and insights, making it easier to understand the observed results from a computational viewpoint.

8. Too many passive sentences are in this article.

The manuscript has been revised to reduce passive sentences and improve clarity.

9. The conclusions must be More Specific to the obtained results. The present conclusions are more general.

We have revised the conclusion to be more specific to the obtained results, focusing on the key findings and their implications. We clearly state the pros and cons of the proposed method. (lines 717-765)

10. The authors should write the practical applications and advantages of this study.

We have outlined the practical applications and advantages of our study in the conclusion in lines 736-748.

Minor issues:

Abstract:

Page 1

Line 19: "matter," It seems that an unnecessary comma. Consider removing the comma.

We have changed the wording (line 16)

Line 28: "simulation," It appears that you have an unnecessary comma in a compound predicate. Consider removing it.

We have changed the wording and made sure there is no unnecessary comma. The corresponding phrase is in line 28 and line 29.

Line 29: "Mosaic" There seems to be a problem with article usage here.

We have changed the wording. The corresponding phrase is in lines 26 and 27.

Introduction:

Page 2

Line 43: "to fully capture its complexity" The to-infinitive to capture has been split by the modifier fully. Avoiding split infinitives can help your writing sound more formal.

We have revised the sentence to avoid the split infinitive. "To fully capture its complexity" has been replaced with "to capture its full complexity," as shown in line 43.

Line 45: "microscopy" It seems that you are missing a comma. Consider adding a comma.

We have added the necessary comma after "microscopy" as indicated in line 45.

Line 49: It appears that starting may be unnecessary in this sentence. Consider removing it

The sentence "often starting from around one billion voxels" has been changed to "often around one billion voxels," as shown in line 50.

Line 52: It appears that the preposition in is redundant. Consider removing it.

We have removed the redundant preposition "in" from the sentence, as shown in line 53.

Page 3

Line 58: It seems that LBM based is missing a hyphen. Consider adding the hyphen(s).

Thank you; we have added the necessary hyphen, as indicated in line 58.

Line 60: It seems that the verb require does not agree with the subject. Consider changing the verb form.

We have changed the structure of the sentence, as indicated in lines 59-61.

Line 61: It seems that real time is missing a hyphen. Consider adding the hyphen(s).

Thank you; we have added the necessary hyphens, as indicated in line 62.

Line 67: The word especially is often overused. Consider using a more specific synonym to improve the sharpness of your writing.

We have changed the sentence “especially by adjusting diffusional conductance coefficients for connected pairs of primitives” to “…particularly in optimizing transport (diffusion) by adjusting diffusional conductance coefficients between connected primitives,” as shown in lines 67-68.

Line 72: "voxels," It seems that you have an unnecessary comma. Consider removing the comma.

We have removed the unnecessary comma after "voxels" as indicated in line 80.

Page 3

Line 91: "(100% saturation)," It appears that you have an unnecessary comma in a compound object. Consider removing it.

We have changed the wording; changes are in lines 94 and 95.

Page 6

Line 138: The word apply appears repeatedly in this text. Consider using a synonym in its place.

We have changed the word apply with employ in line 159 of the revised manuscript.

Line 160: "diffusion" It seems that there is an article usage problem here.

we added the definite article “the” before diffusion in line 160.

Page 7

Line 162: The phrase is equal to maybe wordy. Consider changing the wording.

We reformulated the sentence and removed the phrase” is equal to”. The changes are made in lines 177 and 178.

Page 11

Line 242: The phrase “stands out as” may be wordy. Consider changing the wording.

Thank you for pointing this issue. We have reformulated the whole sentence. The corresponding changes are now in supplementary materials S1 in lines 944-947.

Page 12s

Line 256: The word respire doesn't seem to fit this context. Consider replacing it with a different one.

We have change it to “breath” in line 214.

Page 15

Line 305: This sentence "as well as" may contain incorrect or awkward phrasing. Consider changing it.

We have simply replaced it with “and” in line 265.

Page 16

Line 331: "then" It seems that you are missing a comma. Consider adding a comma.

We have revised the entire sentence and added necessary commas for improved clarity in lines 288–290.

Line 332: "S1" It seems that there is an article usage problem here.

We use now S3A and S3B to cite the flowchart of supplementary materials S2. Changes are in line 292.

Page 17

Line 339: The word “involves” appears repeatedly in this text. Consider using a synonym in its place.

We changed "that involves a large..." to "defined by a large...". The changes are made in line 298 or the revised manuscript.

Line 349: "and" It seems that you are missing a comma. Consider adding a comma.

We have revised the entire sentence; please see lines 307 to 311.

Page 18

Line 365: It appears that your sentence or clause uses an incorrect form of the verb resulted. Consider changing it.

The sentence has been revised, in lines 443 and 444, as follows: We replaced “The resulted segmented 3D binary…” with “The segmentation reveals visible porosities of 17% and 8% for soil bulk densities of 1.2 g cm⁻³ and 1.6 g cm⁻³, respectively”.

Line 368: "MATLAB" It seems that there is an article usage problem here.

We have deleted the specification of the software used for visualising the 3D image.

Page 19

Line 382: "Voxel" It seems that there is an article usage problem here.

We added “the” before Voxel in line 476.

Line 385: It appears that you are missing a comma before the coordinating conjunction and in a compound sentence. Consider adding a comma.

We added the needed comma in line 479.

Line 386-387: A knowledgeable audience might find this sentence hard to read. Consider breaking it into two.

We have changed the wording in lines 481 – 483.

Line 400: It appears that your sentence or clause uses an incorrect form of the verb distribute. Consider changing it.

We corrected the form of the verb distribute in line 496.

Page 20

Line 406: The phrase in accordance with may be wordy. Consider changing the wording.

We changed the phrase with “following Fick's laws” in lines 501-502.

Line 407: “that we” It seems that there is a pronoun problem here.

We have changed it to “which we” in line 503.

Line 417: It appears that results may be unnecessary in this sentence. Consider removing it.

We have noticed that the whole sentence needs to be improved. Changes have been made in lines 514-516.

Page 21

Line 427: The word larger is often overused. Consider using a more specific synonym to improve the sharpness of your writing.

We have changed the word “larger” with “extended” in line 535.

Line 427: The phrase illustrates a comparison of may be wordy. Consider changing the wording.

We have changed the wording. Changes are in lines 539-540.

Line 432: “method,” It seems that you have an unnecessary comma. Consider removing the comma.

The comma after method has been removed in line 545.

Line 433: “LBM-based” It seems that there is an article usage problem here.

We have added the article “the” before LBM-based approach in line 546.

Line 434: “PNGM-based” It seems that there is an article usage problem here.

We have added the article “the” before PNGM -based approach in line 547.

Page 22

Line 460-461: It seems that LBM based is missing a hyphen. Consider adding the hyphen(s).

We added the missing hyphen in line 576.

Line 461: “diffusion,” It appears that you have an unnecessary comma in a compound object.

Consider removing it.

We removed the comma from line 577.

Line 462: “same” It seems that there is an article usage problem here.

We have added the article “the” before ‘same time step’ in line 578.

Page 34

Line 679: “stochastic” It seems that preposition use may be incorrect here.

We added “of” before stochastic in line 642.

Line 680: “normalized” It seems that there is an article usage problem here.

We added “the” before “normalized” in line 643.

Line 681: “update” It appears that your sentence or clause uses an incorrect form of the verb update. Consider changing it.

We have changed update with updating in line 644.

Page 35

Line 697: The phrase compared to may be wordy. Consider changing the wording.

We have changed the wording. Changes are in lines 660 -661.

Line 698: The phrase Accurate determination of may be wordy. Consider changing the wording.

Page 36

We changed the wording and improved the sentence in lines 661-663.

Line 720: The noun phrase explicit scheme seems to be missing a determiner before it. Consider adding an article.

We added the article ‘the’ added before explicit scheme in line 693.

Line 720: “step,” It appears that you have an unnecessary comma in a compound object. Consider removing it.

We removed the comma from line 694.

Line 726: It appears that from may be unnecessary in this sentence. Consider removing it.

We have changed the wording of the sentence in lines 705-710.

Conclusion:

Page 38

Line 759: The word huge is often overused. Consider using a more specific synonym to improve the sharpness of your writing.

We have changed the wording; the corresponding text is in lines 717-727.

Line 761: “an enhanced” The indefinite article, an, may be redundant when used with the uncountable noun accuracy in your sentence. Consider removing it.

We have changed the wording; the corresponding text is in lines 717-727.

Line 765: It seems that the verb outperform does not agree with the subject. Consider changing the verb form.

We have changed the wording; the corresponding text is in lines 717-727.

Line 769: It appears that hence may be unnecessary in this sentence. Consider removing it.

We have changed the wording; the corresponding text is in lines 719-727.

**Reviewer #3:**

 # Summary of the research

The Klai et al. manuscript presents a modeling approach to simulate the microbial decomposition of organic matter in soil that accounts for pore-scale solute transport by diffusion. The approach assumes a two-phase porous medium (solid and liquid). It uses graph theory to abstract the spatial discretization of the pore space based on 3D computed tomography imaging of a soil sample (512^3 voxels with a size of 24 µm^3). The method uses an adjacency matrix to represent a graph constructed from the connectivity of voxels classified as water-filled pore space. Solute transport is simulated using a simplified mass-transfer representation of diffusion. Microbial transformation of organic matter is simulated by numerically solving a differential equation system at each node of the constructed graph. The manuscript aims at improving the accuracy of soil organic matter turnover simulations and advancing a previously developed pore network geometrical modeling method. The new approach is tested against an accurate but computationally demanding reference method (Lattice Boltzmann). The second part of the manuscript applies the introduced approach to improve the computationally efficient pore network modeling approach.

**# Overall impression**

The presented modeling approach has a high potential to contribute in advancing the prediction of microbial decomposition processes in soil while accounting for pore space heterogeneity. However, I see several specific points for improvement.

weakness of the manuscript is the lack of a comprehensive discussion how the presented work relates to other research in the field.

Related to this issue, the authors missed to clearly point out the addresses research challenge and objectives. At the same time part of the conclusion appears partly overreaching. I think the mathematical presentation of the approach requires substantial improvements. Similarly several critical conceptual points require clarification (see details below). The results and discussion section contains a significant methodological part related to parameter estimation required for the application of the new approach with pore network modeling. While I appreciate the attempt to demonstrate the application of the newly developed modeling approach, this introduces a new story with a second methodology (machine learning) to the manuscript that could be better presented more thoroughly in a separate manuscript. Presenting two weakly related stories in one manuscript leads to a rather long manuscript (38 pages) and a relatively high number of figures (15). In addition some detailed quite technical information could be avoided to make the manuscript more concise.

Overall, I think the manuscript presents an innovative approach but requires substantial reorganization and improvement. With a thorough revision it can provide a substantial methodological contribution to advance simulations of microbial decomposition of organic matter in soils.

**# Specific points for improvement**

\## Abstract

The abstract in principle points out the methodological approach nicely. What I missed was a clear statement on the aim and objectives of the study and also a conclusive statement how this approach advances the research field. So I strongly suggest to clearly point out the research challenge at the beginning. I think the statement on the generality of the model (l 21-22) is not substantiated in the paper and overreaching as the presented approach is limited to a two phase systems. As a minor point, the authors use a number of abbreviations that are not properly defined in a self-contained abstract (LBioS, Mosaic models and LBM in l 25,28).

We have revised the abstract to clearly state the aim and objectives of the study. We also highlight that our model efficiently and accurately simulates microbial decomposition of organic matter in soil. These updates can be found in lines 16-25 of the revised manuscript.

We clarified that the model is specifically designed for two-phase systems in line 21. Additionally, we provided definition of the abbreviations, "Mosaic" and "LBioS" in lines 26 to 28, and “VGA” in line 20.

\## Introduction

Current modeling approaches are introduced together with their limitations. However, the authors missed to include a highly relevant and directly related approach (Zech et al. 2022, <https://doi.org/10.1016/j.geoderma.2021.115394>). This paper coupled the cited approach of Portell et al. (2018) with a cellular automata model. While the approach of Zech et al. (2022) is limited to 2D, it is highly relevant approach which numerically solves a PDE-based reaction-diffusion equation system within the pore space derived from computed tomography imaging.

Thank you for pointing out this relevant reference. We have added a discussion on Zech et al. (2022), highlighting how their work coupled diffusion with a microbial decomposition model in a PDE, solved within a 2D pore space derived from computed tomography soil images using the Galerkin method. This work helps set the stage for our contribution to providing a scalable 3D solution in lines 71-77.

The authors claim in the introduction that their method improves current approaches (l 71-72) and requires no calibration (l 73). Instead of anticipating results in the introduction, clarification is needed to outline which research gap(s) are tackled by what objectives.

Thank you for this feedback. We have revised the end of the introduction and removed anticipated results, as reflected in lines 84-90.

As in the abstract, the second part of the study is outlined (l 85ff and l 30ff). This part appears as unconnected to the main study which is also evident from the used phrases "Furthermore" (l30) and "In addition" (l 85) in the text.

We understand this concern and have restructured the introduction to better align this aspect with the main study. We now emphasize that the framework for improving PNGM simulations is a direct extension of our voxel-based approach, as the data was generated by this approach, as reflected in lines 81-83.

\## Material and methods

\### Major issues

The statement on the applicability of the method for unsaturated soil, i.e. to a three-phase system (solid, liquid, air), needs to be clarified by explaining in more detail what "after a method of drainage" means (l 105-106). Only citing previous paper is insufficient for readers.
Although the model is suited for three-phase system (see in Monga et al., 2008), we decided to remove this part as it is not central to the VGA method and simply acknowledge fully saturated conditions (lines 94-95). However, we expanded the explanation to provide a clearer understanding of what means the "method of drainage" in lines 108-115 and includes necessary references for that.

Introducing Fick's law (l 142-155) should be significantly shortened as it is textbook knowledge. The derivation of the equations is hard to follow and should be reorganized (section 2.2.2). Throughout the section, the authors mix differential equations with discretized versions of the equations.

We have revised this section and condensed the knowledge about Fick’s law, we also moved non-essential details (numerical schemes) to the supplementary material S1.

The corresponding changes are in lines 168-172.

To my understanding, the authors applied an operator splitting approach (c.f l 312-314) to numerically solve a diffusion-reaction partial differential equation (cf. Zech et al. 2022, <https://doi.org/10.1016/j.geoderma.2021.115394>). If so it should be clearly indicated and derived based on a presentation of the partial differential equation system.

We did not use a diffusion-reaction partial differential equation. Instead, we applied two different discrete mathematical models. The first is based on Fick’s law, which models diffusion in the graph of connected voxels (Eq 6 or 7 in lines 199 and 201). The second model is an ordinary nonlinear differential equation, solved numerically using both asynchronous and synchronous schemes (flowchart S3A and S3B). Although it is an approach that splits dynamics, it is a model that was derived directly in the graph of voxels—no prior partial differential equation was used (figure 3 in line 149 illustrates the framework).

In addition, estimates on the accuracy loss due to the chosen numerical approximation scheme should be provided. In my understanding the spatial discretization of the diffusion equation is fixed according to the resolution of the computed tomography image (24 µm^3), which should be clearly pointed out to readers.

Thank you for your comment. We confirm that the spatial discretization is fixed according to the computed tomography image resolution (24 µm³), and the accuracy of our numerical approximation is influenced by this resolution.

We clarified that the same framework can be applied at different resolutions, as long as the parameters are adjusted based on the voxel size, which depends on the image resolution. In the revised manuscript, we have mentioned this point more clearly in the limitations of the VGA, which can be found in lines 772-781.

The manuscript provides an Eigenvalue decomposition of the spatially disretized ODE system (l 187 - 202), but fails to point out the purpose of this and what the transformed equations are used for.

Thank you for the comment. The eigenvalue decomposition is a minor point to the main focus of the article. Therefore, we have decided to omit it from the revised manuscript.

Section 2.2.3 points out the temporal discretization of eq. 1 using simple implicit and explicit schemes in a high level of detail that is not essential to understand the paper. Thus, it could be moved to supplementary material. A major point that arises is that for solving ODE systems much more advanced schemes are available. It is unclear why the authors do not use advanced methods as implemented in state-of-the-art ODE solvers. Also, the authors provide an analytical solution to eq. 1 (l 191) which could have been used directly and it became not clear to me why the equation needed to be solved numerically

Thank you for the comment. We have moved the detailed discussion on temporal discretization to the supplementary material S1, as it is not essential to the paper's core focus. The choice to use simple implicit and explicit schemes was made primarily for practical application purposes, to make the framework more straightforward to implement. While more sophisticated numerical methods (adaptative time steps…) could indeed improve computational efficiency. These schemes could be hard to implement due to the size of the pore space, up to hundreds of millions of voxels.

Moreover, directly using the analytical solution for simulations is complex because it requires calculating the exponential of a large matrix, introducing additional computational difficulties.

Indeed, the equation of diffusion is the following

$\frac{dM\left( t \right)}{dt}=-D_{DOM}.\Delta M\left( t \right)$	(S1-2)

Where $M\left( t \right)=\left( m_{1}\left( t \right)...m_{n}\left( t \right) \right)$ and $\Delta=\left( \delta_{i,j} \right)_{1\leq i,j\leq n}$ is the Laplacian matrix of the graph $G\left( E,N \right)$ defined by:

$$\left\{ \begin{aligned} \begin{matrix} \delta_{i,j}=deg\left( i \right) & ifi=j \end{matrix} \\ \begin{matrix} \delta_{i,j}=-1 & ifi\neq j\wedge\left( i,j \right)\in E \end{matrix} \\ \begin{matrix} \delta_{i,j}=0 & otherwise \end{matrix} \end{aligned} \right.$$

where $deg\left( i \right)=\sum_{j:\left( i,j \right)\in E} 1$ is the number of adjacent nodes to the node i.

the equation S1-2 is an ordinary differential equation with analytical solution:

$$M\left( t \right)=e^{-D_{DOM}.t.\Delta}.M_{0}$$

To use this solution, we need to calculate the exponential of a large sparse matrix,

The matrix exponential $e^{-D_{DOM}.t.\Delta}$ of the matrix $-D_{DOM}.t.\Delta$ is computed as an infinite series:

$$I-D_{DOM}.t.\Delta+\frac{\left( D_{DOM}.t.\Delta\right)^{2}}{2!}-\frac{\left( D_{DOM}.t.\Delta\right)^{3}}{3!}+\ldots$$

Unfortunately, sparse matrices contain many zero elements, and efficient storage and computation leverage this sparsity. Our adjacency matrix $\Delta$ (of size tenth of millions, in the specific dataset of this study: 23 million) is very sparse as most nodes are only connected to a few other nodes (6 connected nodes as maximum, since we are using a 6-connectivity policy): the multiplication of sparse matrices often results in matrices with more non-zero elements, this is because new connections or interactions can emerge between previously disconnected nodes.

As higher powers of the matrix are computed (e.g., $\Delta^{2},\Delta^{3},\Delta^{4},\Delta^{5}$), the result becomes denser, leading to a rapid increase in memory and computational demands. Since the cost of calculating the analytical solution is high, we use Euler schemes to discretize the time derivative. However, there exist many approaches and tools capable of calculating the exponential of large sparse matrices like those found in MATLAB and SciPy. The existing methods can hardly be used, in practice, for matrices of size up to 20 million.

The same explanation is now included in lines 886- 908 of the S1 Appendix, in of the revised manuscript.

The governing equation describing microbial dynamics (l 270) indicates that growth respiration is not considered, which is in contrast to the fact that microbial catabolism’s and anabolism are coupled ,typically represented in microbially explicit models by yield coefficients (c.f. Eq. 1 in Manzoni et al. 2009, [https://doi.org/10.1016/j.soilbio.2009.02.031](https://doi.org/10.1016/j.soilbio.2009.02.031%20) ).

Thank you for this in-depth comment. As stated in Vogel et al., 2015, we followed the approach of the SOMKO model (Gignoux et al., 2001) and MIOR model (Masse et al., 2007) where microbes are subjected to 3 physiological processess: survival, maintenance and growth. In this approach, CO2 flux is computed as a linear function of biomass. This approach considers offer-demand issue: indeed, in case of an offer of C too low to sustain maintenance, growth (demand) is not fulfilled. As reported in the review of Manzoni & Porporato (2009) and raised by the Reviewer, most of the C models assume growth respiration proportional to uptake (yield or carbon use efficiency CUE approach). In these models, growth respiration is thus not included in the carbon mass balance to consider offer-demand issue. In more complex approaches, biosynthesis and CO2 flux are calculated from the stoechiometry of chemical reactions (e.g. Ginovart et al., 2005).

This information is included now within the text in lines 248-251.

It is unclear what "system 3" refers to (l 328) and what's the rationale to apply an asynchronous scheme to solve the reactive ODE system at each graph node (l 329-331) as a coupled ODE system must be solved for all coupled state variables simultaneously to achieve correct results.

The "system 3", mentioned by the reviewer, refers to the system of equations (12) in line 266. The rationale for using the asynchronous scheme for transformation processes (S3A and S3B) lies in the challenges associated with nonlinearity in solving the discrete (in space) Eq. 11 on the discretized pore space, represented as a graph of voxels. This setup requires a very small-time step to avoid negative values. The synchronous and asynchronous schemes provide a stable approach to simulating the dynamics described in Eq. 11 while allowing parallel computations across the nodes of the graph.

Indeed globally, the aim of the present paper is to present computational methods which are realistic to apply for huge data such as 3D micro-CT images of soil samples (up to 3000x3000x3000 sizes). The main interest is to be able running simulations by taking into account a fine spatialization of the dynamics.

We added in lines 993-996 the following text: “Flowcharts S3A and S3B provide a stable algorithm to approximate dynamics of equation (12), by tolerating the use of reasonable time steps. In contrast, applying drastically the explicit scheme (synchronous scheme) of transformation (equation 13) requires using very small-time steps”.

The authors claim that the spatial discretization of the model, which is given by the pixel size of 24 µm, is suited to resolve microbial activity and simulate diffusive transport (l 357-359). It would be important to provide evidence for this claim.

However, for simulating diffusion a spatial discretization of 24 µm could be too coarse.

In addition, a large part of the pores are not resolved by computed tomography imaging (visible porosity is only 8 and 17%, l 365-366) and pores smaller than 24 µm are potentially relevant for microbial activity. Also, the effective resolution of the image at a pixel size of 24 µm would be rather 50 µm assuming that at least two pixels must be considered to clearly differentiate between water-filled pores and solid matrix. So, it would be important to at least clearly point out the issue of unresolved pore space as a limitation of the approach.

We acknowledge the reviewer’s concern regarding the resolution of the computed tomography (CT) image. The 24 µm voxel size was chosen based on the balance between available data and computational feasibility. Although finer pores smaller than 24 µm are not resolved by this imaging technique, and such pores could indeed be significant for microbial processes, the model still provides meaningful insights into diffusive transport and microbial interactions at this scale. We also emphasize that the visible porosity detected in the segmented 3D image aligns with values reported in similar studies like Monga et al 2022 and Mbé et al 2021, and the simulated microbial activity focuses on the pore space that is resolved. While a finer resolution would certainly provide more detail, it would also lead to an increase in computational demand and might require different modelling strategies. However, the computational efficiency of our method would allow to process data with higher resolution.

We have added in the limitations of our method, in lines 750-752, that its accuracy is influenced by the spatial discretization, determined by the voxel resolution.

###Minor issues

The equation numbering is incomplete, some equations are numbered, some are not.

Subsection 1.2.3.2 does not exist; this is probably a typo (l 343).

Thank you for pointing that out. We have reviewed the manuscript and corrected the numbring of equations in whole text. Additionally, we have addressed the typo in line 302 about subsection numbering; it should correctly refer to supplementary materials S1.

\## Results and discussion

In a first part, the section essentially shows the comparison of simulations with the new approach with the other two approaches. In addition, results from numerical tests. A second part introduces an approach and show its results to improve PNGM modelling approaches. I'd recommend to focus on the first part and add a discussion of own results in relation to other experimental and modelling studies in the research field. It is the major weakness of the manuscript that such a discussion is lacking.

Another important issue that I see with the results/ discussion section is that it is overloaded with details on material and methods, which should be shifted in the corresponding section (see details below).

We moved the theoretical aspects of improving PNGM to materials and methods section starting from line 315 to line 425.

The Results and Discussion section now focuses on comparing the new approach with the other two methods and discussing an application of the theoretical framework for approximating diffusional conductance coefficients.

\### Major issues

For the pore network geometrical modelling approach a general coefficient alpha=0.6 was used (l 407) and a rationale for selecting this value would be needed for readers.

The selection of the value 0.6 for alpha was chosen to maximize the intercorrelation between the curves of PNGM and LBM. The calibration process using intercorrelation maximization is described now in material and methods section in lines 365 – 373.

A specific value for D_DOM was used (l 458-459). A rationale or derivation of the value is not provided, but would be important as this parameter probably affects microbial organic matter turnover by diffusion-limitations of DOC.

The same parameter value for $D_{DOM}$ was used across all three methods for consistency in comparison purposes. The value used correspond to the diffusion coefficient of an organic molecule in water .

This value has been used in many investigations (Vogel et al 2015, Monga et al 2014, 2022, Mbé et al 2022 ..)

We added in lines 570-571 the following text: “The value used corresponds to the diffusion coefficient of an organic molecule in water [34]. We added the reference

“Rumble J, editor. CRC handbook of chemistry and physics.” in line 878.

This includes also the derivation of the unit (voxels^2 j-1). What unit is "j"? I'd expect a diffusion coefficent given in a unit of [length^2]/[time] (e.g., µm^2/h).

The unit "j" was a typographical error in the manuscript, stemming from the French term “jour,” meaning day. We have corrected it to the appropriate unit. (line 570).

For the voxel unit, 1 voxel = 24 µm.

For me it is also unclear what the authors mean by "with all other diffusion processes of other compounds canceled".

We apologize for the oversight in the initial draft. Initially, we modelled the transport of microorganisms, CO₂, and dissolved organic matter (DOM). However, we later focused solely on the transport of DOM, aligning with the approaches used in studies such as Monga et al. and Mbé et al. (2022).

We deleted the unnecessary sentence “with all other diffusion processes of other compounds canceled” from line 592.
