# Supplementary material for: A voxel-based approach for simulating microbial decomposition in soil: Comparison with LBM and improvement of morphological models
Source: PLoS One. 2025 Mar 3;20(3):e0313853. doi: 10.1371/journal.pone.0313853 (PMC11875386; doi:10.1371/journal.pone.0313853)
Supplement: Attachment — Submitted filename: Response to Reviewers.docx [file pone.0313853.s004.docx]

Review Comments to the Author

Please use the space provided to explain your answers to the questions above. You may also include additional comments for the author, including concerns about dual publication, research ethics, or publication ethics. (Please upload your review as an attachment if it exceeds 20,000 characters)

Reviewer #1: This study presents a novel computational method for simulating microbial decomposition in soil using 3D micro-CT images. It uses a valuated graph of connected voxels to model complex diffusion and transformation processes. The method includes parallelization strategies and evaluates various numerical techniques. Validation against LBioS (lattice-Boltzmann method) and Mosaic (Pore Network Geometrical Modelling) shows it matches LBM-based simulation accuracy with a quarter of the computing time and exceeds Mosaic in accuracy without calibration. Additionally, it enhances PNGM-based simulations by approximating diffusional conductance coefficients using stochastic gradient descent.

Based on the initial assessment of this manuscript, the current decision is a major revision to reconsider the publication with Plos One.

Abstract1. Line 18-19: It would be good to point out the research gap before running into aims at the beginning of your abstract (be concise, please; after all, it is just an abstract)

We added a few sentences describing the research gap.

.2. Line 25: Could you please define LbioS in your abstract as it first appears in your content?

We defined Lbios within the abstract

3. Line 28: one-fourth -> a quarter

We replaced “one-fourth” by “a quarter” and add a sentence pointed out that the computing time decrease can be a key point.

4.

Line 33: It would be better if you could restate/highlight your engineering contribution from the novel method you developed in this study (please be concise; after all, it is just an abstract).Introduction

The last sentences has been reduced to be more concise but we keep one last sentence dealing with the computation of diffusive conductive coefficients. The reason is that this promising way becomes much more easy to investigate due to the computing time gain.

5. Line 51: These efforts aim to … ? or These studies aim to … ? Can efforts aim to do something?

We replaced “these efforts aim to…” by a more adequate sentence.

6. Line 68: You have not accomplished your literature review by identifying a research gap in high clarity. You have not compared and contrasted the pros and cons from the historical track of previous studies (the initial, later progress, and state of the art by now; what has been done already and what else has not been achieved yet?) in order to identify those research gaps before you propose your research objectives, followed by methods from Line 69. Your review of published works should solidly support your research purposes before heading into it. There is a significant gap or logical disconnection between Lines 68 and 69.

We agree about the need to better highlight the research gap and improve the logical flow between the literature review and our proposed objectives. We have restructured the literature review to more clearly contrast previous studies, identify existing gaps, and establish the rationale for our proposed research objectives and methods

7. Lines 82-87: You have structurally illustrated your paper in the second last paragraph without mentioning the specific sections/chapters. You don’t need to redo it with an independent paragraph that looks slightly redundant.

We agree that the redundancy should be avoided. We have combined the structural overview into a single paragraph and removed the redundant content.

8. At the end of your Introduction and right after your research proposal, you should re-highlight your research significance and engineering contributions to dazzle the reviewers and readers, who have short memories after reading a series of lengthy content, because not all of them can easily recall all the content you provided in the background and literature review in the Introduction. Note that it must be concise and informative.

We have revised the conclusion of the Introduction to re-emphasize our research’s significance and engineering contributions in a concise and impactful manner, ensuring that the key points remain fresh in the reader's mind.

Material and methods

9. Lines 91-92: You can just write, “In this study, we only considered the condition that soil is fully saturated, i.e., soil pore space is filled with water, in exception that soil under the unsaturated condition, i.e., soil void space is partially filled with soil moisture.” An aquatic environment could represent many other meanings, not only in topsoil, vadose zone, and groundwater under the phreatic line (i.e., groundwater table) but also in other water bodies, e.g., nearshore seawater, surface flow, etc.

Thank you for the clarification. We have revised the sentence as suggested to more accurately describe the fully saturated soil condition while avoiding ambiguity regarding the term "aquatic environment."

10. For a numerical contribution like this, I recommend you relocate your pseudo code (algorithm) into an appendix and leave a Matlab or Python code in your supplementary file so many researchers interested in this code will further work on it. It would be much better if you could draw a flow chart for your pseudo-code as a figure in the main text rather than in the appendix and supplementary material.

We appreciate your suggestion. We have replaced the pseudocode with a flowchart, which we believe makes the process more readable and visually intuitive. Additionally, in section 2.5 (Implementation Details), we have included a link (<https://github.com/mouadklai/VGA_microbial_decomposition>) to the C implementation for further reference.

11. Since it was not observable in your figures in terms of a few very important key points for a numerical simulation, e.g., boundary conditions, initial conditions, etc., you should better visualize those in your simulation demo figures and also provide other information regarding convergence and numerical stability issues (e.g., size of discretization, temporal step in your implicit and explicit schemes, simulating scale compared to single particles or the representativeness of REV, etc.) in the numerical method you just developed. Those will help you defend your work from other peer reviewers who are experts in developing numerical schemes. Results and discussion

Thank you for highlighting the importance of including more details regarding boundary conditions, initial conditions, and key numerical aspects such as discretization size, time-stepping, and representativeness.

Boundary Conditions: These are now detailed in subsection 2.5.3 of the paper.

Initial Conditions: These are explained in the Results and Discussion section, where each simulation for diffusion and microbial activity is compared.

Stability and Convergence: Stability results are discussed in the Results and Discussion section. However, we have added explicit details about numerical stability and convergence in subsection 2.2.3 on numerical schemes for the graph diffusion equation.

Briefly:

Explicit Scheme: Easier to implement and computationally cheaper but requires smaller time steps to maintain stability.

Implicit Scheme: More stable with respect to time step size but involves solving linear systems at each time step.

12. As you used image analysis on CT scans, have you provided adequate technical details on CT scanning and image processing, e.g., voxel-length calibration, ratio, measuring precision, etc.?

Thank you for your valuable comment. We appreciate the need for more information regarding the dataset used for the simulations and how it was processed. we provided additional context and detail regarding the 3D microcomputed tomography (µCT) image data, soil characteristics, and the segmentation techniques applied. It can be found in section 2.6 dataset description and it included a new subsection about boundary conditions

13. Line 516: You claimed you adopted the D3Q9 scheme for LBM simulation. So far, to the best of my knowledge, it should have D2Q9, D3Q15, and D3Q27. Could you please visualize your D3Q9 cell in 3D space, as you demonstrated in Figure 5? Please don’t forget to update all figure numbers in both their captions and content.

Thank you for the feedback. It is true that, by mistake, we referenced the D3Q9 scheme instead of D3Q19. We updated our manuscript accordingly. Below is a figure illustrating the D3Q19 scheme used in this study, sourced from: https://link.springer.com/article/10.1007/s11242-011-9914-7.

14. As for many diffusion coefficients or diffusivities you adopted in your numerical simulations, I recommend you defend your selection of those by comparing them against published works, i.e., necessary citations of new references should be added in. This question could also be raised to select other parameters of the properties of fluid and porous media in your work. It will convince experts who have more experience in this area.

Thank you for your feedback regarding the selection of diffusion coefficients and other parameters. The diffusion coefficients and other biological parameters used in our simulations were adopted from previous works to remain within a similar range of experimentally relevant values. These parameters were chosen based on their practical applicability and relevance to our study, reflecting their experimental nature.

We acknowledge that the exact values of diffusion coefficients can vary; however, our simulations demonstrate that the overall trends and results remain consistent regardless of the specific diffusion coefficient used. This consistency suggests that the main findings are robust and not overly dependent on the precise value of the diffusion coefficient. We clarified this point in the revised manuscript and discuss in section 3 how our results are representative of general behaviour rather than being sensitive to specific parameter values.

15. Lines 734-754: The pros and cons could be better summarized in a table for comprehensive comparison and contrast. You can also do so to compare different numerical methods. It will shorten the content you must deliver to readers and make the paper more concise and informative, i.e., straightforward to read and check all essentials in just a few tables. Conclusions

Thank you for your insightful suggestion regarding the organization of the content in lines 734-754. We agree that summarizing the pros and cons, as well as comparing different numerical methods in a table format, would enhance the readability and conciseness of our paper.

In response to your suggestion, we have included a table that clearly outlines the pros and cons of the different approaches, providing a comprehensive comparison.

16. Please reconstruct your concluding section as follows:Paragraph 1: Concise summary of your research gaps identified through literature review and research objectives; Briefing your methodology with some very critical settings;Paragraph 2: Listing your conclusions or research findings point-by-point;Paragraph 3: Mention the limitations and reflection of your work; Highlight the significance of your research and engineering applications (this should be done at the end of your Introduction as well because it will substantially enhance your academic significance, i.e., self-advertising).References

Thank you for your valuable feedback. We revised the conclusion to address your comments by summarizing the research gaps and objectives, briefly describing the methodology. We then listed the findings and contributions in a point-by-point format, and concluded with a discussion of the limitations and the significance of the research, including its practical applications.

17. The reference list you provided is inconsistent; e.g., some have the year in brackets ahead while others are at the end with even the month. Please sort those out and get them fixed according to the PloS One standard.Figures and Quality

Thank you for pointing out the inconsistencies in the reference list. We have corrected the formatting to align with the PLoS ONE standard.

18. All figures are provided in very blurry forms, resulting in difficulty in reading and checking all essential details. It could be an issue caused by Word-to-PDF transformation. Moreover, despite Figures 1-5, the font sizes of values in axes, labels, and legends in all other figures are too small to be read; e.g., the axis label “z” is missed in Figure 6 without any unit and interpretation.19. Last but not least, please scrutinize your manuscript using grammar software (e.g., Grammarly, Writefull, Quiltbot, etc.) to screen out all grammatical mistakes and typos before the subsequent submission of your revised draft.

Thank you for your feedback regarding the figures. We have changes figures with their best quality. Also, we have corrected figure 6 to include the missing axis label “z”

Reviewer #2: The authors investigated a voxel-based approach for simulating microbial decomposition in soil: comparison with LBM and improvement of morphological models. Before the publication of this paper, I have raised some issues as follows:

Major issues:

1. The authors should improve the Abstract of the study.

We have updated the abstract to make it clearer and more concise, clearly stating the research challenge, objectives, and contributions of our study.

2. The authors should revise the Introduction of the study by including the novelty.

We have revised the introduction to clearly highlight the novelty of our approach.

3. What was the basis for choosing the mentioned ranges for the investigated parameters?

The ranges for the investigated parameters were based on a previous study comparing PNGM and LBM (Monga et al. (2022)). These parameters were selected to match those used in that comparison, ensuring that our focus was on comparing overall simulation results rather than affecting the comparison itself.

4. The authors should thoroughly clarify each concept with physical significance.

We have included detailed explanations of the underlying physical principles.

5. A comparison is expected with the experimental results.

We agree that comparing our results with experimental data is important. This paper primarily aims to evaluate our simulation method against existing approaches. The model used in this study was validated by experimental results from Monga et al. (2014), as cited in subsection 2.3 of the text.

6. When utilizing articles in sentences, authors should be more careful.

Thank you for pointing this out. We have carefully reviewed and revised the use of articles in sentences usage throughout the manuscript.

7. A reasonable physical explanation should be provided for the observed trends, not only to report what is graphically seen in the figures. More physical insight into the Discussion section is needed.

The results now include more physical explanations to provide better context and understanding of the observed trends.

8. Too many passive sentences are in this article.

The manuscript has been revised to reduce passive sentences and improve clarity.

9. The conclusions must be More Specific to the obtained results. The present conclusions are more general.

We have revised the conclusion to be more specific to the obtained results, focusing on the key findings and their implications.

10. The authors should write the practical applications and advantages of this study.

We have a section outlining the practical applications and advantages of our study.

Minor issues:

Abstract:Page 1Line 19: "matter," It seems that an unnecessary comma. Consider removing the comma.

We removed the unnecessary comma after "matter."

Line 28: "simulation," It appears that you have an unnecessary comma in a compound predicate. Consider removing it.

We removed the extra comma in the compound predicate.

Line 29: "Mosaic" There seems to be a problem with article usage here.

We removed the extra comma

Introduction :

Page 2

Line 43: "to fully capture its complexity" The to-infinitive to capture has been split by the modifier fully. Avoiding split infinitives can help your writing sound more formal.

We have revised the sentence to avoid the split infinitive: "to fully capture its complexity" is replaced by “ to capture its full complexity”

Line 45: "microscopy" It seems that you are missing a comma. Consider adding a comma.

We have added the necessary comma after "microscopy."

Line 49: It appears that starting may be unnecessary in this sentence. Consider removing it

The sentence “often starting from around one billion voxels” has been changed to “often around one billion voxels”

Line 52: It appears that the preposition in is redundant. Consider removing it.

We have removed the redundant preposition "in" from the sentence.

Page 3

Line 58: It seems that LBM based is missing a hyphen. Consider adding the hyphen(s).

Thank you, we have added the needed hyphens.

Line 60: It seems that the verb require does not agree with the subject. Consider changing the verb form.

We have changed the structure of the sentence

Line 61: It seems that real time is missing a hyphen. Consider adding the hyphen(s).

Thank you, we have added the needed hyphens.

Line 67: The word especially is often overused. Consider using a more specific synonym to improve the sharpness of your writing.

We have changed the sentence
“especially by adjusting diffusional conductance coefficients for connected pairs of primitives”

To

“ particularly in optimizing transport by adjusting diffusional conductance coefficients between connected primitives”

Line 72: "voxels," It seems that you have an unnecessary comma. Consider removing the comma.

We have removed the unnecessary comma after "voxels."

Page 3

Line 91: "(100% saturation)," It appears that you have an unnecessary comma in a compound object. Consider removing it.

We have removed the unnecessary comma.

Page 6

Line 138: The word apply appears repeatedly in this text. Consider using a synonym in its place.

We have changed it to employ

Line 160: "diffusion" It seems that there is an article usage problem here.

The discussion is about a specific diffusion process (the diffusion process of DOM). we added the definite article “the” .

Page 7

Line 162: The phrase is equal to maybe wordy. Consider changing the wording.

We reformulated the sentence and removed the phrase is equal

Page 11

Line 242: The phrase stands out as may be wordy. Consider changing the wording.

Thank you for pointing this issue. We have reformulated the hole sentence.

Page 12

Line 256: The word respire doesn't seem to fit this context. Consider replacing it with a different one.

We have change it to “breath”

Page 15

Line 305: This sentence "as well as" may contain incorrect or awkward phrasing. Consider changing it.

We have simply replaced it with “and”.

Page 16

Line 331: "then" It seems that you are missing a comma. Consider adding a comma.

We have added a comma

We have changed the hole sentence with necessary commas for more clarity.

Line 332: "S1" It seems that there is an article usage problem here.

We use now S1A and S2B to cite the flowchart in the appendix

Page 17

Line 339: The word involves appears repeatedly in this text. Consider using a synonym in its place.

We changed the repeated verb “appears”

Line 349: "and" It seems that you are missing a comma. Consider adding a comma.

We added a comma before “and”

Page 18

Line 365: It appears that your sentence or clause uses an incorrect form of the verb resulted. Consider changing it.

The sentence has been revised as follows:

" The segmentation reveals … ” rather than “The resulted segmented 3D binary”

Line 368: "MATLAB" It seems that there is an article usage problem here.

We have deleted the specification of the software.

Page 19

Line 382: "Voxel" It seems that there is an article usage problem here.

We added “the” before Voxel

Line 385: It appears that you are missing a comma before the coordinating conjunction and in a compound sentence. Consider adding a comma.

We added the needed comma

Line 386-387: A knowledgeable audience might find this sentence hard to read. Consider breaking it into two.

We have changed it

Line 400: It appears that your sentence or clause uses an incorrect form of the verb distribute. Consider changing it.

We corrected the form of the verb

Page 20

Line 406: The phrase in accordance with may be wordy. Consider changing the wording.

We changed to “following Fick's laws”

Line 407: “that we” It seems that there is a pronoun problem here.

We have changed it to “which we”

Line 417: It appears that results may be unnecessary in this sentence. Consider removing it.

We have noticed that the whole sentence needs to be improved, thank you

Page 21

Line 427: The word larger is often overused. Consider using a more specific synonym to improve the sharpness of your writing.

We have reduced the use of the word “larger”

Line 427: The phrase illustrates a comparison of may be wordy. Consider changing the wording.

We have changed the wording.

Line 432: “method,” It seems that you have an unnecessary comma. Consider removing the comma.

The comma has been removed

Line 433: “LBM-based” It seems that there is an article usage problem here.

We have added the article “the” before LBM-based approach

Line 434: “PNGM-based” It seems that there is an article usage problem here.

We have added the article “the” before PNGM -based approach

Page 22

Line 460-461: It seems that LBM based is missing a hyphen. Consider adding the hyphen(s).

We added hyphen.

Line 461: “diffusion,” It appears that you have an unnecessary comma in a compound object.

Consider removing it.

We removed comma.

Line 462: “same” It seems that there is an article usage problem here.

We have added the article “the” before ‘same time step’

Page 34

Line 679: “stochastic” It seems that preposition use may be incorrect here.

We added “of” before stochastic

Line 680: “normalized” It seems that there is an article usage problem here.

We added “the” before “normalized”

Line 681: “update” It appears that your sentence or clause uses an incorrect form of the verb update. Consider changing it.

We changed to updating

Page 35

Line 697: The phrase compared to may be wordy. Consider changing the wording.

We have changed the wording

Line 698: The phrase Accurate determination of may be wordy. Consider changing the wording.

Page 36

We changed the wording and improved the sentence.

Line 720: The noun phrase explicit scheme seems to be missing a determiner before it. Consider adding an article.

We added the article ‘the’ added before explicit scheme

Line 720: “step,” It appears that you have an unnecessary comma in a compound object. Consider removing it.

We removed the comma removed.

Line 726: It appears that from may be unnecessary in this sentence. Consider removing it.

We removed “that”.

Conclusion:

Page 38

Line 759: The word huge is often overused. Consider using a more specific synonym to improve the sharpness of your writing.

We have changed the word “huge” by “substantial”

Line 761: “an enhanced” The indefinite article, an, may be redundant when used with the uncountable noun accuracy in your sentence. Consider removing it.

The indefinite article “an” has been removed

Line 765: It seems that the verb outperform does not agree with the subject. Consider changing the verb form.

The verb has been corrected to ensure subject-verb agreement.

Line 769: It appears that hence may be unnecessary in this sentence. Consider removing it.

The word “hence” has been removed to simplify the sentence

**Reviewer #3:**

 # Summary of the research

The Klai et al. manuscript presents a modeling approach to simulate the microbial decomposition of organic matter in soil that accounts for pore-scale solute transport by diffusion. The approach assumes a two-phase porous medium (solid and liquid). It uses graph theory to abstract the spatial discretization of the pore space based on 3D computed tomography imaging of a soil sample (512^3 voxels with a size of 24 µm^3). The method uses an adjacency matrix to represent a graph constructed from the connectivity of voxels classified as water-filled pore space. Solute transport is simulated using a simplified mass-transfer representation of diffusion. Microbial transformation of organic matter is simulated by numerically solving a differential equation system at each node of the constructed graph. The manuscript aims at improving the accuracy of soil organic matter turnover simulations and advancing a previously developed pore network geometrical modeling method. The new approach is tested against an accurate but computationally demanding reference method (Lattice Boltzmann). The second part of the manuscript applies the introduced approach to improve the computationally efficient pore network modeling approach.

**# Overall impression**

The presented modeling appraoch has a high potential to contribute in advancing the prediction of microbial decomposition processes in soil while accounting for pore space heterogeneity. However, I see several specific points for improvement.

weakness of the manuscript is the lack of a comprehensive discussion how the presented work relates to other research in the field.

Related to this issue, the authors missed to clearly point out the addresses research challenge and objectives. At the same time part of the conclusion appears partly overreaching. I think the mathematical presentation of the approach requires substantial improvements. Similarly several critical conceptual points require clarification (see details below). The results and discussion section contains a significant methodological part related to parameter estimation required for the application of the new approach with pore network modeling. While I appreciate the attempt to demonstrate the application of the newly developed modeling approach, this introduces a new story with a second methodology (machine learning) to the manuscript that could be better presented more thoroughly in a separate manuscript. Presenting two weakly related stories in one manuscript leads to a rather long manuscript (38 pages) and a relatively high number of figures (15). In addition some detailed quite technical information could be avoided to make the manuscript more concise.

Overall, I think the manuscript presents an innovative approach but requires substantial reorganization and improvement. With a thorough revision it can provide a substantial methodological contribution to advance simulations of microbial decomposition of organic matter in soils.

**# Specific points for improvement**

\## Abstract

The abstract in principle points out the methodological approach nicely. What I missed was a clear statement on the aim and objectives of the study and also a conclusive statement how this approach advances the research field. So I strongly suggest to clearly point out the research challenge at the beginning. I think the statement on the generality of the model (l 21-22) is not substantiated in the paper and overreaching as the presented approach is limited to a two phase systems. As a minor point, the authors use a number of abbreviations that are not properly defined in a self-contained abstract (LBioS, Mosaic models and LBM in l 25,28).

We have revised the abstract to clearly state the aim and objectives of the study. We now explicitly mention that our model addresses the challenge of efficiently and accurately simulating microbial decomposition in complex soil matrices. We have also clarified that the model is specifically designed for two-phase systems, addressing the concern about the generality of our approach. Additionally, we added key points about terms "Mosaic" and "LBioS".

\## Introduction

Current modeling approaches are introduced together with their limitations. However, the authors missed to include a highly relevant and directly related approach (Zech et al. 2022, <https://doi.org/10.1016/j.geoderma.2021.115394>). This paper coupled the cited approach of Portell et al. (2018) with a cellular automata model. While the approach of Zech et al. (2022) is limited to 2D, it is highly relevant approach which numerically solves a PDE-based reaction-diffussion equation system within the pore space derived from computed tomography imaging.

Thank you for pointing out this relevant reference. We have added a discussion on Zech et al. (2022), highlighting how their work coupled a lattice Boltzmann method with a microbial decomposition model within a 2D pore space. This addition helps clarify the relevance of their approach and sets the stage for our contribution to providing a scalable 3D solution.

The authors claim in the introduction that their method improves current approaches (l 71-72) and requires no calibration (l 73). Instead of anticipating results in the introduction, clarification is needed to outline which research gap(s) are tackled by what objectives.
Thank you for this feedback. We have revised the introduction to clearly articulate the research gap without anticipating the results. The revised text now focuses on the limitations of existing methods, such as the computational cost of LBM and the calibration needs of PNGM, and then transitions smoothly to our proposed solution without introducing results prematurely.

As in the abstract, the second part of the study is outlined (l 85ff and l 30ff). This part appears as unconnected to the main study which is also evident from the used phrases "Furthermore" (l30) and "In addition" (l 85) in the text.

We understand this concern and have restructured the introduction to better align this aspect with the main study. We now emphasize that the framework for improving PNGM simulations is a direct extension of our voxel-based approach, as data was generated by the main study.

\## Material and methods

\### Major issues

The statement on the applicability of the method for unsaturated soil, i.e. to a three-phase system (soilid, liquid, air), needs to be clarified by explaining in more detail what "after a method of drainage" means (l 105-106). Only citing previous paper is insufficent for readers.

We agree that the original statement lacked sufficient detail. We have now expanded the explanation to provide a clearer understanding of what the "method of drainage"

Introducing Fick's law (l 142-155) should be significantly shortened as it is textbook knowledge. The derivation of the equations is hard to follow and should be reorganized (section 2.2.2). Throughout the section, the authors mix differential equations with dicretized versions of the equations.

We have revised this section and condensed the knowledge about Fick’s law, we also moved non-essential details (numerical schemes) to the supplementary material.

To my understanding, the authors applied an operator splitting approach (c.f l 312-314) to numerically solve a diffusion-reaction partial differential equation (cf. Zech et al. 2022, <https://doi.org/10.1016/j.geoderma.2021.115394>). If so it should be clearly indicated and derived based on a presentation of the partial differential equation system.

We did not use a diffusion-reaction partial differential equation. Instead, we applied two different discrete mathematical models. The first is based on Fick’s law, which models diffusion in a graph of connected voxels. The second model is an ordinary nonlinear differential equation, solved numerically using both asynchronous and synchronous schemes. Although it is a dynamics splitting approach, it was derived directly from the image data—no prior partial differential equation was used. The modelling process started directly on the voxels of the image.

In addition, estimates on the accuracy loss due to the chosen numerical approximation scheme should be provided. In my understanding the spatial discretization of the diffusion equation is fixed according to the resolution of the computed tomography image (24 µm^3), which should be clearly pointed out to readers.

Thank you for your comment. We confirm that the spatial discretization is fixed according to the computed tomography image resolution (24 µm³). The accuracy of our numerical approximation is influenced by this resolution.

We clarified that the same framework can be applied at different resolutions, as long as the parameters are adjusted based on the voxel size, which depends on the image resolution. In the revised manuscript, we have mentioned this point more clearly.

The manuscript provides an Eigenvalue decomposition of the spatially disretized ODE system (l 187 - 202), but fails to point out the purpose of this and what the transformed equations are used for.

Thank you for the comment. The eigenvalue decomposition is not essential to the main focus of the article. Therefore, we have decided to omit it from the revised manuscript.

Section 2.2.3 points out the temporal discretization of eq. 1 using simple implicit and explicit schemes in a high level of detail that is not essential to understand the paper. Thus it could be moved to supplementary material. A major point that arises is that for solving ODE systems much more advanced schemes are available. It is unclear why the authors do not use advanced methods as implemented in state-of-the-art ODE solvers. Also, the authors provide an analytical solution to eq. 1 (l 191) which could have been used directly and it became not clear to me why the equation needed to be solved numerically

Thank you for the comment. We have moved the detailed discussion on temporal discretization (Section 2.2.3) to the supplementary material, as it is not essential to the paper's core focus. The choice to use simple implicit and explicit schemes was made primarily for practical application purposes, to make the framework more straightforward to implement. While more sophisticated numerical methods (adaptative time steps…) could indeed improve computational efficiency. These schemes could be hard to implement due to the size of the pore space, up to hundreds millions of voxels. Moreover, directly using the analytical solution for simulations is complex because it would need to be coupled with the transformation processes at each simulation step which is not straightforward. While using the analytical solution would be preferable for diffusion alone, its application becomes challenging in our context, as it requires calculating the exponential of a large matrix, introducing additional computational difficulties. Given that this level of mathematical detail is not central to the paper's main objectives, we have chosen to omit it in the revised manuscript.

The governing equation describing microbial dynamics (l 270) indicates that growth respiration is not considered, which is in contrast to the fact that microbial catabolism’s and anabolism are coupled ,typically represented in microbially explicit models by yield coefficients (c.f. Eq. 1 in Manzoni et al. 2009, [https://doi.org/10.1016/j.soilbio.2009.02.031](https://doi.org/10.1016/j.soilbio.2009.02.031%20) ).

Growth respiration is considered but it is decoupled from growth Monod equation, as we did not use a yield coefficient as already explained in Vogel et al 2015. This also allows to not fixing a constant value for the yield coefficient which is actually highly debated.’

It is unclear what "system 3" refers to (l 328) and what's the rationale to apply an asynchronous scheme to solve the reactive ODE system at each graph node (l 329-331) as a coupled ODE system must be solved for all coupled state variables simultaneously to achieve correct results.

The "system 3", mentioned by the reviewer, refers to the system of equations labelled as Eq. 3 in Section 3.4. The rationale for using the advanced algorithms we term as synchronous and asynchronous schemes (S2A and S2B) lies in the challenges associated with solving the discrete nonlinear Eq. 3 on the discretized pore space, represented as a graph of voxels. This setup requires a very small-time step to avoid negative values. The synchronous and asynchronous schemes provide a stable approach to simulating the dynamics described in Eq. 3 while allowing parallel computations across the nodes of the graph.

Indeed globally, the aim of the present paper is to present computational methods which are realistic to apply for huge data such as 3D micro-CT images of soil samples (up to 3000x3000x3000 sizes). The main interest is to be able running simulations by taking into account a fine spatialization of the dynamics.

The authors claim that the spatial discretization of the model, which is given by the pixel size of 24 µm, is suited to resolve microbial activity and simulate diffusive transport (l 357-359). It would be important to provide evidence for this claim.

However, for simulating diffusion a spatial discretization of 24 µm could be too coarse.

In addition, a large part of the pores are not resolved by computed tomography imaging (visible porosity is only 8 and 17%, l 365-366) and pores smaller than 24 µm are potentially relevant for microbial activity. Also, the effective resolution of the image at a pixel size of 24 µm would be rather 50 µm assuming that at least two pixels must be considered to clearly differentiate between water-filled pores and solid matrix. So, it would be important to at least clearly point out the issue of unresolved pore space as a limitation of the approach.

We acknowledge the reviewer’s concern regarding the resolution of the computed tomography (CT) image. The 24 µm voxel size was chosen based on the balance between available data and computational feasibility. Although finer pores smaller than 24 µm are not resolved by this imaging technique, and such pores could indeed be significant for microbial processes, the model still provides meaningful insights into diffusive transport and microbial interactions at this scale. We also emphasize that the visible porosity detected in the segmented 3D image aligns with values reported in similar studies like Monga et al 2022 and Mbé et al 2021, and the simulated microbial activity focuses on the pore space that is resolved. While a higher resolution would certainly provide more detail, it would also lead to an increase in computational demand and might require different modelling strategies. However, the computational efficiency of our method would allow to process data with higher resolution.

###Minor issues

The equation numbering is incomplete, some equations are numbered, some are not.

Subsection 1.2.3.2 does not exist, this is probably a typo (l 343).

Thank you for pointing that out. We have reviewed the manuscript and corrected the equation. Additionally, we have addressed the typo in subsection numbering; it should correctly refer to supplementary materials S1.

\## Results and discussion

In a first part, the section essentially shows the comparison of simulations with the new approach with the other two approaches. In addition, results from numerical tests. A second part introduces an approach and show its results to improve PNGM modelling approaches. I'd recommend to focus on the first part and add a discussion of own results in relation to other experimental and modelling studies in the research field. It is the major weakness of the manuscript that such a discussion is lacking.

Another important issue that I see with the results/ discussion section is that it is overloaded with details on material and methods, which should be shifted in the corresponding section (see details below).

We moved the theoretical aspects of improving PNGM and additional details on materials and methods to the corresponding section. The Results and Discussion section now focuses on comparing the new approach with the other two methods and discussing the application example.

\### Major issues

For the pore network geometrical modelling approach a general coefficient alpha=0.6 was used (l 407) and a rationale for selecting this value would be needed for readers.

The selection of the value 0.6 for alpha is that it maximize the intercorrelation between the curves of PNGM and LBM. The calibration process using intercorrelation maximization is described now in material and methods section in subsection 2.4.

A specific value for D_DOM was used (l 458-459). A rationale or derivation of the value is not provided, but would be important as this parameter probably affects microbial organic matter turnover by diffusion-limitations of DOC.

The same parameter value for $D_{DOM}$ was used across all three methods for consistency in comparison purposes. We did not provide a specific rationale or derivation for this value in the manuscript. However, we acknowledge that this parameter can influence microbial organic matter turnover through diffusion limitations of dissolved organic carbon

This includes also the derivation of the unit (voxels^2 j-1). What unit is "j"? I'd expect a diffusion coefficent given in a unit of [length^2]/[time] (e.g., µm^2/h).

The unit "j" was a typographical error in the manuscript, stemming from the French term “jour,” meaning day. We have corrected it to the appropriate unit. For voxel units, we have adjusted the dimensions to reflect that 1 voxel corresponds to a rectangular surface area of 24 x 24 micrometers.

For me it is also unclear what the authors mean by "with all other diffusion processes of other compounds canceled".

We apologize for the oversight in the initial draft. Initially, we modelled the transport of microorganisms, CO₂, and dissolved organic matter (DOM). However, we later focused solely on the transport of DOM, aligning with the approaches used in studies such as Monga et al. and Mbé et al. (2022). The selection of parameter ranges was intended for comparative purposes with existing methods. We have removed the sentence.

Several tests using explicit and implicit schemes with different time steps are reported in quite some detail (e.g., table 3 and l 479-487). I'd suggest to only report the optimal variant in the main manuscript and move the detailed results to the supplementary material.

The presented result take a lot of space (text and figures) in the manuscript but will be not of high interest for most readers.

The supplementary tests on time steps has been moved to supplementary materials S2 Appendix

From the presented test results, I also had the impression that the numerical schemes were not the optimal solution. The simple explicit Euler scheme is known to produce low-accuracy results for two large time steps. A time step of 0.1 s was selected as the largest possible (l 483). Could you provide an estimate of the accuracy of the numerical solution? Alternatively more advanced schemes could be used, including adaptive time stepping.

Our decision to use simple implicit and explicit schemes was guided primarily by the aim to keep the framework practical and easy to implement. While we acknowledge that more advanced numerical methods could enhance computational efficiency, our focus was on creating a model that is accessible and straightforward to reproduce. Regarding the accuracy, we selected a time step of 0.1 s as it provided a good balance between stability and computational cost, but we recognize that this may not be optimal for all cases. Although we did not conduct a formal accuracy analysis, we agree that incorporating adaptive time-stepping or more sophisticated schemes could offer improved accuracy and efficiency in future developments.

The diffusion conductance coefficients approximation is introduced very detailed but difficult to grasp (section 3.3). Also, it is mostly not a result but a method description. The text uses a notation from machine learning (e.g. training instead of calibration) inconstantly.

The part about approximating the diffusional conductance coefficients has been moved to materials and methods section and only results of the application example is still in the results and discussion section.

For instance, it is indicated that a neural network is used (l 588-589), which is to my understanding not correct and if so more details on the structure of the neural network should be provided.

We apologize for the confusion. We meant to say a simple machine learning approach, not a simple neural network.

The stochastic gradient descent method is introduced very detailed (l 611-649), which I would consider as supplementary information not of high interest for most readers. While the authors describe the method, a reference to the source of the information is missing and would be important to include.

Thank you for pointing out this issue, we have reduced the details about SGD

I disagree with the claimed improvement in accuracy of the new PNGM model ( l 722-723). From Figure 15 it is evident that the results from both PNGM model variants are very close and both are not matching well with the VGA simulations.

We have tried to be more realistic, we said that: While the "new PNGM" model maintains the structural characteristics of the "old PNGM" model, we acknowledge that it only slightly improved the model’s performance.

While section 3.4 indicates a to provide a discussion on limitations of the approach, I could not find it in the text. In contrast, the listed advantages appear overreaching.

For instance, the indicated use of neural networks for ubscaling (l 748-752) is rather a potential future approach then a direct outcome of the study (=advantage). Regarding the generality (l 753-754), it would be particularly import to discuss limitations of the presented approach with respect to the representation of microbial community dynamics, spatial discretization (determined by voxel resolution), restriction to a two-phase system and advective transport.

In the new conclusion we discuss the advantages as well as he limitations of our approach. Thank you for pointing out this issue.

\### Minor issues

Missing reference [35] in l 440.

We changed reference [35] by [30].

For my understanding two abbreviations are used for the same approach: VGA=GDE, right?

Better use only one (e.g. l 510-512)."more" instead of "much" accurate representation (l 515)?

GDE is for graph diffusion equation that is used in the Voxel Graph-based Approach (VGA)

\# Conclusion

The conclusion partly repeats section 3.4. I'd suggest to merge both. What is also missing is a link to other approaches (e.g., Zech et al. 2022, Zech et al. 2022, <https://doi.org/10.1016/j.geoderma.2021.115394>) to clearly point out where this approach advances the research field.

We merged the content of Section 3.4 with the conclusion in order to avoid repetition and streamline the discussion. In the revised conclusion, we clearly outline how our Voxel Graph-based Approach (VGA) advances the research field by linking it to other recent methods, including those by Pot et al. (2022), Monga et al. (2022), and Zech et al. (2022).

6. PLOS authors have the option to publish the peer review history of their article ([what does this mean?](https://secureurl.ankabut.ac.ae/fmlurlsvc/?fewReq=:B:JVYyOT07Mi5+NTomOC5hbDU4OTI4OS57YW9maXx9em01Oms4PD09MDtuPTptbW09ajk5bmluMW1rOjs4Pzw6bWowajAwbT9pbC58NTk/Ojk7PTE/PjwueWFsNTw+QjtZUWFbODk9PDAwJTw+QjtZUWFdODk9PDAwLnpreHw1ZWdgaWVtbCZiZ31hZmFIY30maWsmaW0uazU7OC5gbGQ1OA==&url=https%3a%2f%2fjournals.plos.org%2fplosone%2fs%2feditorial-and-peer-review-process%23loc-peer-review-history)). If published, this will include your full peer review and any attached files.


If you choose “no”, your identity will remain anonymous but your review may still be made public.


**Do you want your identity to be public for this peer review?** For information about this choice, including consent withdrawal, please see our [Privacy Policy](https://secureurl.ankabut.ac.ae/fmlurlsvc/?fewReq=:B:JVYyOT07Mi5+NTomOC5hbDU4OTI4OS57YW9maXx9em01Pj9tPzhsPz07MGw+MW47MTE+bDBsaj48bm09OTw7OjE9ajk5PDBqbS58NTk/Ojk7PTE/PjwueWFsNTw+QjtZUWFbODk9PDAwJTw+QjtZUWFdODk9PDAwLnpreHw1ZWdgaWVtbCZiZ31hZmFIY30maWsmaW0uazU7OC5gbGQ1OA==&url=https%3a%2f%2fwww.plos.org%2fprivacy-policy).

Reviewer #1: NoReviewer #2: NoReviewer #3: No

[NOTE: If reviewer comments were submitted as an attachment file, they will be attached to this email and accessible via the submission site. Please log into your account, locate the manuscript record, and check for the action link "View Attachments". If this link does not appear, there are no attachment files.]

While revising your submission, please upload your figure files to the Preflight Analysis and Conversion Engine (PACE) digital diagnostic tool, [https://pacev2.apexcovantage.com/](https://secureurl.ankabut.ac.ae/fmlurlsvc/?fewReq=:B:JVYyOT07Mi5+NTomOC5hbDU4OTI4OS57YW9maXx9em01PTAxbDk9aTg9OzE4azoxMT4wajkwPm47azw6MDttbWo6bDxtOmxtPC58NTk/Ojk7PTE/PjwueWFsNTw+QjtZUWFbODk9PDAwJTw+QjtZUWFdODk9PDAwLnpreHw1ZWdgaWVtbCZiZ31hZmFIY30maWsmaW0uazU9Oi5gbGQ1OA==&url=https%3a%2f%2fpacev2.apexcovantage.com%2f). PACE helps ensure that figures meet PLOS requirements. To use PACE, you must first register as a user. Registration is free. Then, login and navigate to the UPLOAD tab, where you will find detailed instructions on how to use the tool. If you encounter any issues or have any questions when using PACE, please email PLOS at [figures@plos.org](mailto:figures@plos.org). Please note that Supporting Information files do not need this step.

*In compliance with data protection regulations, you may request that we remove your personal registration details at any time.* [*(Remove my information/details)*](https://secureurl.ankabut.ac.ae/fmlurlsvc/?fewReq=:B:JVYyOT07Mi5+NTomOC5hbDU4OTI4OS57YW9maXx9em01MWs9PGxubTltPjBtOD08aW49OGlqP2o5bDE9OWw+MDo+PDAwajExPy58NTk/Ojk7PTE/PjwueWFsNTw+QjtZUWFbODk9PDAwJTw+QjtZUWFdODk9PDAwLnpreHw1ZWdgaWVtbCZiZ31hZmFIY30maWsmaW0uazU7OC5gbGQ1OA==&url=https%3a%2f%2fwww.editorialmanager.com%2fpone%2flogin.asp%3fa%3dr)*. Please contact the publication office if you have any questions.*
